# Papel do Ecocardiograma na Avaliação do Septo Interatrial e Pesquisa de Forame Oval Patente como Fonte Emboligênica

**DOI:** 10.36660/abc.20220903

**Published:** 2023-10-16

**Authors:** Angele Azevedo Alves Mattoso, Joberto Pinheiro Sena, Viviane Tiemi Hotta

**Affiliations:** 1 Hospital Santa Izabel Salvador BA Brasil Hospital Santa Izabel, Salvador, BA – Brasil; 2 Hospital Santa Izabel Salvador BA Brasil Hospital Santa Izabel – Hemodinâmica, Salvador, BA – Brasil; 3 Instituto do Coração HC FMUSP São Paulo SP Brasil Instituto do Coração HC-FMUSP – Unidade Clinica de Miocardiopatias e Doenças da Aorta, São Paulo, SP – Brasil; 4 Fleury Medicina e Saúde São Paulo SP Brasil Fleury Medicina e Saúde, São Paulo, SP – Brasil

**Keywords:** Ecocardiografia, Septo Atrial, Forame Oval Patente

## Abstract

A comunicação do septo atrial (CIA) representa, aproximadamente, de 6%-10% dos defeitos cardíacos congênitos, com incidência de 1 em 1.500 nascidos vivos.^1^ Forame oval patente (FOP) é mais comum e está presente em mais de 20%-25% dos adultos.^2^ Síndromes clínicas associadas a CIA e FOP são variáveis, com implicações abrangendo a medicina pediátrica e adulta, neurologia e cirurgia.

O interesse adicional na anatomia do septo interatrial (SIA) aumentou substancialmente nas últimas duas décadas, com evolução simultânea dos procedimentos percutâneos envolvendo cardiopatia estrutural do lado esquerdo e procedimentos eletrofisiológicos. Idealmente, essas intervenções baseadas em cateter requerem rota direta para o átrio esquerdo (AE) através do SIA, necessitando completo entendimento de sua anatomia. Atualmente, tecnologias de imagem sofisticadas e não invasivas como ecocardiografia transesofágica bidimensional (ETE 2D) e tridimensional (ETE 3D), ressonância cardíaca (RMC) e tomografia computadorizada (TC) passaram por um extraordinário desenvolvimento tecnológico, fornecendo detalhes anatômicos das estruturas cardíacas visualizadas em formato 2D e 3D e são essenciais para diagnóstico e tratamento de pacientes com doenças cardíacas.

A avaliação da anatomia e anormalidades do SIA, portanto, requer abordagem padronizada e sistemática, integrando modalidades diagnósticas e fornecendo avaliação adequada e uniforme para terapias cirúrgicas e transcateter.

## Figura Central

**Figure:**
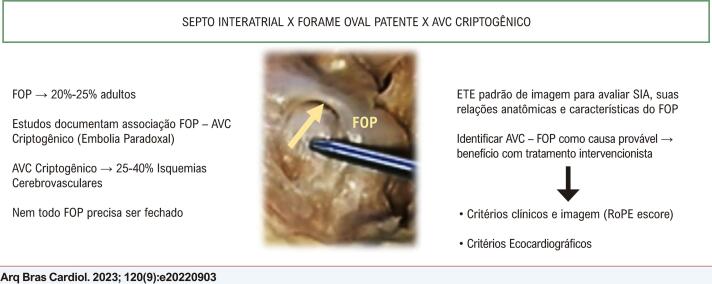
: Papel do Ecocardiograma na Avaliação do Septo Interatrial e Pesquisa de Forame Oval Patente como Fonte Emboligênica Imagem cedida pela Dra Vera Demarchi Aiello, Laboratório de Anatomia Patológica do Instituto do Coração do Hospital das Clínicas da FMUSP.

## Anatomia do septo interatrial

O SIA tem três componentes: septo primum (SP), septo secundum (SS) e septo do canal atrioventricular. O seio venoso não é um componente do SIA verdadeiro, mas sim uma estrutura adjacente, através da qual pode ocorrer uma comunicação atrial.^3,4^

**Figura 1:**
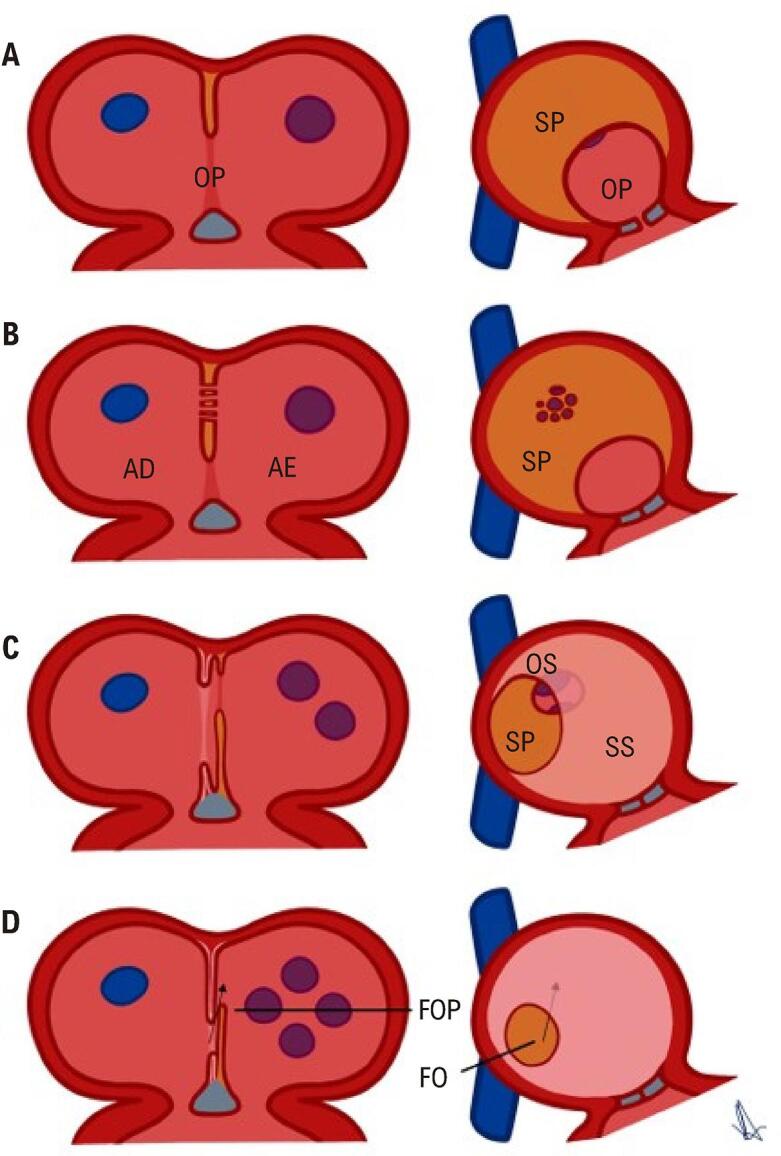
– Esquema demonstrando o desenvolvimento normal do SIA. A) SP cresce a partir do teto atrial. B) Fenestrações se desenvolvem dentro do SP. C) SS desenvolve-se por dobradura da parede atrial. O óstio secundum atua como conduto para passagem direita-esquerda de sangue oxigenado. D) Na borda anterossuperior da FO, o SP e o SS permanecem não fundidos, o que constitui o FOP. A seta denota sangue fluindo através do FOP do AD embrionário para o AE. Os pontos azuis e roxos representam o desenvolvimento do influxo das veias cavas e pulmonares para os átrios. CE: coxim endocárdico; FO: fossa oval; FOP: forame oval patente; OP: óstio primum; OS: óstio secundum; SAI: septo interatrial; SP: septo primum; SS: septo secundum.

Os átrios se desenvolvem primeiro como cavidade comum. Em aproximadamente 28 dias de gestação, o SP, oriundo do teto atrial, começa migrar em direção aos coxins endocárdicos em desenvolvimento. Durante essa transição, o espaço entre o SP e o coxim endocárdico é denominado “óstio primum embrionário” ou “forame primum” (Figura 1A, 1B).

**Figura 2:**
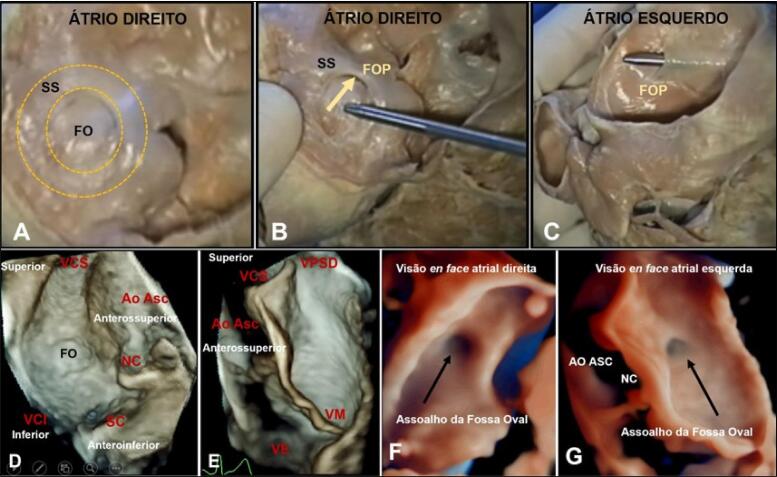
– Espécime anatômico do SIA de perspectiva atrial direita (A, B) e atrial esquerda (C) mostrando SP e SS não completamente fundidos, o que constitui o FOP. A seta denota o pertuito por onde o sangue flui através do FOP do AD para o AE, cortesia de Dra Vera Demarchi Aiello, Laboratório de Anatomia Patológica do Instituto do Coração do Hospital das Clínicas da FMUSP. Imagens ecocardiográficas transesofágicas tridimensionais (modo 3D Zoom) do SIA e estruturas adjacentes. Visões (modo 3D Zoom) en face ou frontal do SIA de perspectiva atrial direita (D) e atrial esquerda (E), respectivamente. (F, G) Visões “en face” (modo 3D Zoom – True Vue) do SIA mostrando o assoalho da FO (seta). Ao Asc: aorta ascendente; FO: fossa oval; FOP: forame oval patente; SC: seio coronariano; NC: seio aórtico não coronariano; SP: septo primum; SS: septo secundum; VCI: veia cava inferior; VCS: veia cava superior; VM: valva mitral; VPSD: veia pulmonar superior direita.

O SS desenvolve-se à direita do SP, estendendo-se superior, posterior e inferiormente e é resultado de dobradura do teto atrial em vez de estrutura membranosa verdadeira (Figura 1C); Sobreposição do SP no lado atrial esquerdo torna-se o assoalho de depressão ovalada (fossa oval), delimitada pela borda resultante da dobradura SS, vista no lado atrial direito. O óstio primum se fecha pela fusão das células mesenquimais do SP com coxins endocárdicos. Aos dois meses de gestação, o SS e o SP se fundem, deixando o forame oval como única comunicação residual (Figura 1D). O SP funciona como válvula de retalho na vida fetal, direcionando o fluxo sanguíneo do átrio direito (AD) para AE através do óstio secundum ou forame oval, sendo essencial para fornecer sangue oxigenado da placenta para órgãos vitais. Após o nascimento, essa abertura se fecha quando pressão mais alta no AE empurra o *flap* da válvula contra a borda muscular. Se a adesão estiver incompleta em toda a margem do rebordo, permanece o FOP (Figura 2A, 2B, 2C).

Quando observado por perspectiva atrial direita, o SIA aparece como extensa área, delimitada inferior e superiormente pelos orifícios da veia cava inferior (VCI) e veia cava superior (VCS), respectivamente; anteroinferiormente, esta superfície é limitada pelo orifício do seio coronário, anterossuperiormente, pelo seio aórtico não-coronário e por fim, anteriormente pela região de implante da cúspide da valva tricúspide. A ETE 3D é a melhor modalidade de imagem para descrever essa superfície e suas relações anatômicas, de perspectivas atrial esquerda e direita com precisão comparável ao de espécimes anatômicos (Figura 2D, 2E, 2F, 2G). Anatomistas descrevem o SIA como uma separação interposta entre AD e AE. Portanto, a punção do SIA cria uma comunicação direta entre os átrios sem invadir tecidos epicárdicos. Todavia, esta definição corresponde, de fato, ao assoalho da fossa oval (FO), que se assemelha a depressão do tipo cratera, quando a face atrial direita do septo é visualizada “*en face* ou frontal” (Figuras 2A, 2B, 2D e 2F) e como parede fina sobreposta do lado atrial esquerdo (Figuras 2C, 2E e 2G).

**Figura 3:**
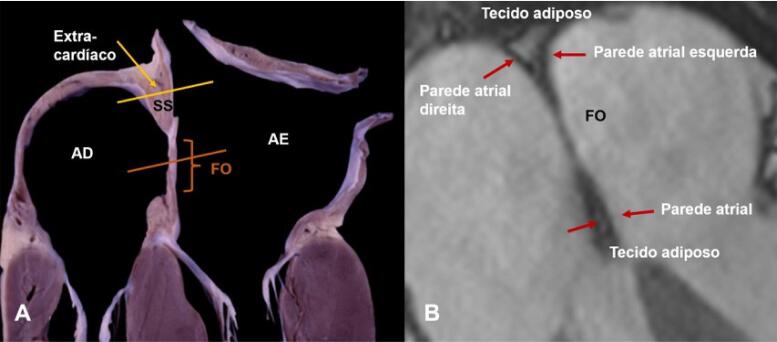
– (A) Espécime anatômico mostrando a área muscular que circunda a FO, interposta entre AD e AE, o ‘’SS’’ ou ‘’limbo da FO‘’, que é uma dobradura da parede atrial. A punção dessa região interna do AD produz saída das cavidades atriais para tecidos adiposos “externos” que ocupam a dobradura (“falso septo”). (B) Imagem transversal de RM obtida em paciente com hipertrofia lipomatosa do SIA, mostrando duas ‘’pseudomassas’’ preenchidas por tecido adiposo localizadas superiormente, contíguas à veia cava superior e inferiormente ao septo atrioventricular. AD: átrio direito; AE: átrio esquerdo; FO: fossa oval; SS: septo secundum.

A FO é, portanto, o SP embrionário, e cobre cerca de 20% da área supracitada, sendo definida como “septo verdadeiro”.^4^ A área muscular que circunda as margens superior, posterior e inferior da FO que à primeira vista parece estar interposta entre os átrios, o ‘’septo secundum ou limbo da FO’’, como vimos, na verdade, não separa realmente AD do AE. A Dissecção dessa área revela que se trata de dobradura da parede atrial e a punção dessa região interna do AD produz saída das cavidades atriais para tecidos adiposos “externos” que ocupam a dobradura. Assim, essa dobradura é, sem dúvida, um “falso septo” (Figura 3).

**Figura 4:**
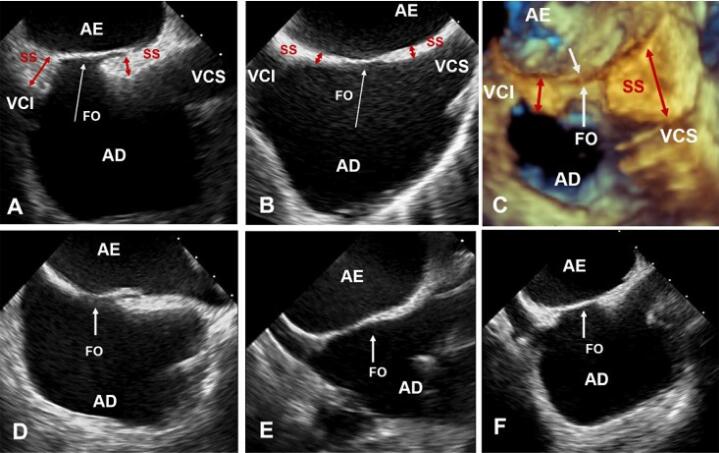
– Imagens ecocardiográficas transesofágicas do SIA através da visão bicaval mostrando diferentes tamanhos de FO. (A, F) Presença de tecido adiposo significativo no SS ou sulco interatrial (setas), dando ao SIA aparência de ampulheta. (B, D, E) Pouco tecido adiposo, tornando o sulco atrial (seta) quase virtual. (C) Significativo acúmulo de tecido adiposo na dobradura interna do septo (invadindo o sulco interatrial ou SS) (setas vermelhas), sem comprometer a lâmina da FO (setas brancas), a chamada hipertrofia lipomatosa. AD: átrio direito; AE: átrio esquerdo; FO: fossa oval; SAI: septo interatrial; SS: septo secundum; VCI: veia cava inferior; VCS: veia cava superior.

Entretanto, para consistência com a literatura, refere-se a dobradura da parede atrial como SS. Corretamente descrita no século 19 por Waterston^5^ e conhecida como sulco interatrial de Sondergaard, essa dobradura é preenchida por quantidade variável de tecido adiposo epicárdico e pequenos vasos. A ETE 2D pode, potencialmente, identificar o tecido adiposo, com aparência característica de ampulheta, mas em indivíduos normais, o sulco interatrial é quase virtual, portanto, difícil distinguir uma pequena quantidade de tecido adiposo entre as duas paredes atriais que se assemelham a parede única. Em outros pacientes, o tecido adiposo é particularmente intrusivo, invadindo o sulco interatrial e separando as paredes atriais (Figura 4).

A TC pode identificar tecido adiposo com base nos limiares de atenuação, entretanto, apesar da alta resolução espacial da técnica, os limites entre o tecido adiposo e a parede atrial permanecem indistintos. A melhor modalidade de imagem para ilustrar essa arquitetura é a ressonância cardíaca (RMC), cuja imagem do septo mantem o mesmo formato 2D que ETE 2D. A RMC permite uma distinção clara entre o tecido muscular e o adiposo (Figura 3B).

**Figura 5:**
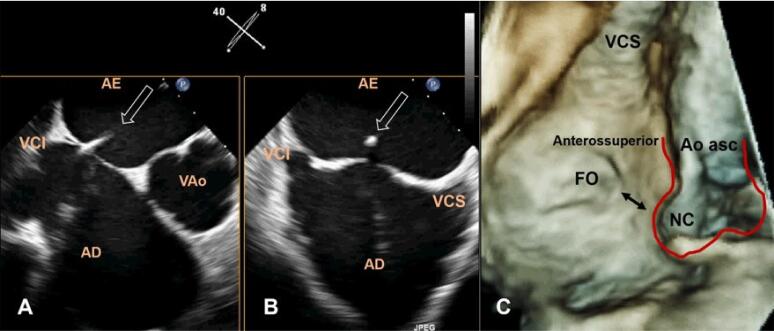
– (A, B) Imagens ETE 3D biplanar (X-plane) guiando a punção transeptal atrial local-específico para procedimento de oclusão percutânea de apêndice atrial esquerdo em posição mais posterior e inferior do septo. A seta indica um cateter de punção bem posicionado. (C) Imagem de ETE 3D en face (perspectiva atrial direita) do SIA mostrando a proximidade (seta dupla) do seio aórtico não-coronariano com a região da FO. Seio aórtico e porção proximal de aorta ascendente delineados. AD: átrio direito; AE: átrio esquerdo; Ao Asc: aorta ascendente; FO: fossa oval; NC: seio aórtico não-coronariano; VAO: valva aórtica; VCI: veia cava inferior; VSC: veia cava superior.

A FO pode ter diferentes tamanhos e localizações, dependendo da extensão variável, tanto do tecido da lâmina, quanto do sulco interatrial, que não é uniforme em toda a FO (Figura 4). Definir o tamanho da FO é importante porque o local mais seguro para punção transeptal (PTS) é através da mesma. Dada a grande variedade de procedimentos em cardiopatia estrutural tratadas por via percutânea, o local da PTS (sempre dentro da FO) varia de acordo com o procedimento realizado (PTS local-específico). Por exemplo, para implantar um dispositivo oclusor de apêndice atrial esquerdo na orientação correta, o operador puncionará o septo em uma posição posterior e inferior, enquanto para procedimento de valva mitral a posição é mais alta (Figura 5A, 5B).

Geralmente, a FO maior está em grandes átrios e a FO menor está associada ao acúmulo de tecido adiposo na dobradura interna, a chamada hipertrofia lipomatosa (Figura 4C).

Outra área de interesse é a região anterosuperior, que confina o seio pericárdico transverso e, através dele, a raiz aórtica, a estrutura mais importante próxima a FO. Alguns milímetros separam a margem anterosuperior da FO da parede atrial direita, que recobre o seio aórtico não coronário. De fato, a complicação mais temida da PTS é a punção da raiz da aorta (Figura 5C).

## Hipertrofia lipomatosa

O termo hipertrofia lipomatosa do SIA, criado por Prior em 1964, é questionável, pois ao contrário do lipoma, o tecido da hipertrofia lipomatosa não é encapsulado e o acúmulo de gordura é caracterizado histologicamente por hiperplasia de adipócitos (número aumentado), não hipertrofia (tamanho aumentado). Esse acúmulo de adipócitos é externo ao coração, preenchendo o sulco interatrial. As Imagens de RMC podem definir a natureza externa desse tecido adiposo (Figura 3B). Embora o acúmulo de tecido adiposo dentro do sulco interatrial (SS) tenha aparência característica de ampulheta na ecocardiografia, tanto ETE 2D quanto 3D (Figura 4) geralmente não conseguem diferenciar claramente o tecido adiposo das paredes atriais. As bordas hipertróficas foram definidas com espessura do SS de 8 mm, enquanto a lipomatosa, com espessura de 15 mm.

A Hipertrofia lipomatosa do SIA é benigna, embora grande quantidade de tecido adiposo possa esporadicamente obstruir a entrada da VCS ou criar distorção das paredes atriais, com consequentes arritmias.

## Bolsa Septal (*Septo Pouch*) e Crista Septal

Anomalias na fusão do SIA podem resultar em variantes anatômicas, incluindo bolsa septal atrial, crista septal atrial esquerda, e anatomia mista que inclui bolsa e crista.^6^

**Figura 6:**
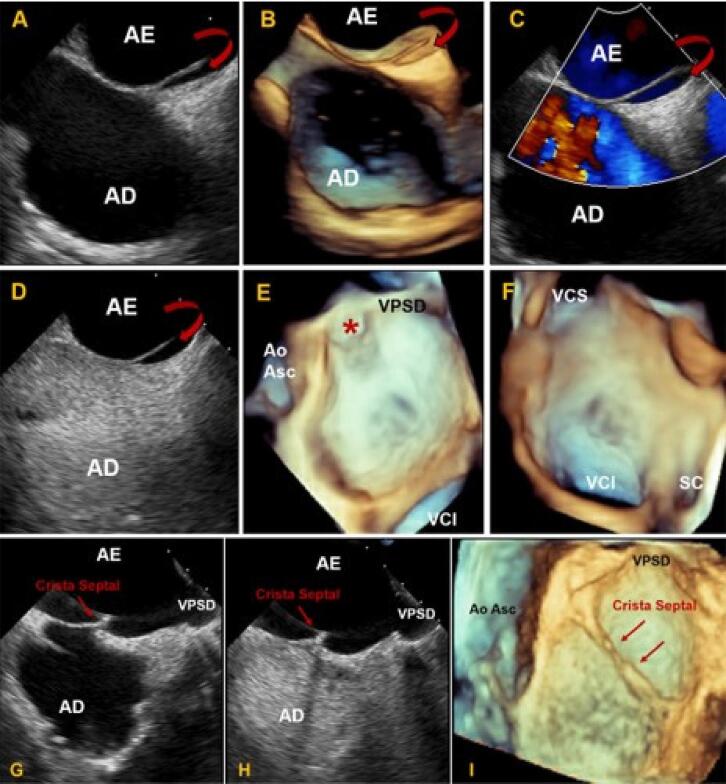
– Imagens ecocardiográficas transesofágicas mostrando estrutura semelhante a bolsa em SIA em visão bicaval bidimensional (A) e tridimensional (B) com o orifício da bolsa septal esquerda (setas curvadas). (C e D) Doppler em cores (C) e uso de microbolhas de salina agitada (D) mostrando ausência de shunt septal. Imagens ecocardiográficas em (E e F) Visão “en face” do SIA, de perspectiva atrial esquerda (E) e direita (F). Imagens ecocardiográficas transesofágicas de SIA com presença de crista septal. (G) Visão bidimensional bicaval mostrando imagem de crista septal (seta). (H) Visão bidimensional bicaval mostrando integridade do SIA durante injeção de contraste de salina agitada. (I) Visão en face (3D Zoom) do SIA de perspectiva atrial esquerda mostrando morfologia ecocardiográfica tridimensional da crista septal (setas). AD: átrio direito; AE: átrio esquerdo; Ao Asc: aorta ascendente; SC: seio coronariano; SAI: septo interatrial; VCI: veia cava inferior; VCS: veia cava superior; VPSD: veia pulmonar superior direita.

Bolsa septal, estrutura semelhante a bolsa de canguru, sem *shunt* interatrial, abre-se com mais frequência no AE, ou, menos comumente, no AD, ocorrendo provavelmente por causa de fusão incompleta entre SP e SS (Figura 6A-6F).

Em 2006, Roberson et al., descreveram malformação do SIA consistindo em SIA duplo com câmara na linha média.^7^ Acredita-se que esta descrição seja consistente com grande bolsa septal. Ainda não está claro, porém tem sido sugerido que o sangue estagnado nesta bolsa pode ser um possível nicho para formação de trombo e embolia.^8,9^ As ETEs 2D e 3D são as melhores técnicas para obter imagens da bolsa septal.

A FO e o sulco interatrial constituem a extensa área que recobre a parede medial do AE. Esta área, com exceção da bolsa septal, é quase desprovida de outras estruturas. Entretanto, embora raramente, a proeminente formação em crista semelhante a membrana ao longo da FO e se estendendo até a parede livre atrial tem sido observada.^10^A crista septal atrial, caracterizada por estrutura tubular no lado atrial esquerdo do SIA, decorrendo ao longo da FO, é uma área de tecido espessada localizada e acredita-se que seja devido à fusão irregular dos septos (Figura 6G-6I). A presença de crista atrial esquerda pode potencialmente interferir na PTS em pacientes submetidos a procedimentos transcateter.

## Forame Oval Patente

**Figura 7:**
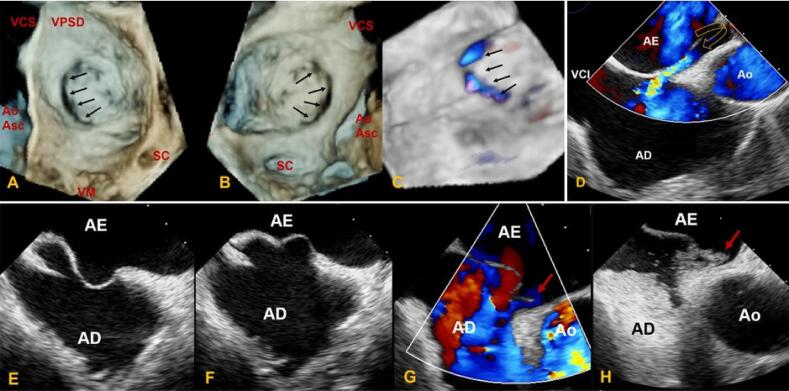
– Visão en face ecocardiográfica transesofágica tridimensional (modo 3D Zoom) do SIA, mostrando o aneurisma da lâmina da FO associado a descontinuidade elíptica (setas) entre os átrios de perspectiva atrial esquerda (A) e direita (B) e confirmada com Doppler em cores (C). (D) Imagem transesofágica bidimensional de FOP ‘‘stretched “ (seta curva) em visão bicaval com fluxo ao Doppler colorido da esquerda para a direita. (E, F) Imagem transesofágica bidimensional do SIA com mobilidade excessiva da lâmina da FO e (G) FOP associado (seta vermelha) demonstrado ao Doppler colorido com fluxo da esquerda para direita e passagem intermitente de microbolhas de salina agitada da direita para esquerda (H). AD: átrio direito; AE: átrio esquerdo; Ao: aorta; Ao Asc: aorta ascendente; FO: fossa oval; SC: seio coronariano; SAI: septo interatrial; VCS: veia cava superior; VCI: veia cava inferior; VM: valva mitral; VPSD: veia pulmonar superior direita.

O FOP não é uma verdadeira deficiência do SIA, pois não há deficiência estrutural do tecido, mas uma separação potencial entre SP e SS, localizada na porção anterosuperior do SIA.^3^ O forame permanece funcionalmente fechado enquanto a pressão do AE for maior que a pressão do AD, podendo ser apenas funcionalmente patente e ter aparência de túnel, com o SP formando uma válvula oscilante (Figura Central e Figura 2). As diferenças relativas nas pressões atriais podem resultar em desvio intermitente de sangue. Entretanto, o FOP também pode ser uma abertura elíptica verdadeira entre os átrios (Figura 7A-7C).

Alguns casos de FOP resultam do “estiramento ou *stretching”* da banda límbica superior do SS por dilatação e remodelamento atrial (Figura 7D). Em outros casos, o SP é verdadeiramente aneurismático, e como tal, não consegue fechar completamente a CIA (Figura 7E-7H). O tamanho do FOP em estudos de autopsia varia de 1 a 19 mm (média de 4,9 mm).^11^

Para fins de nomenclatura, o ‘‘FOP’’ é referido como a passagem de sangue da direita para a esquerda, demonstrado ao Doppler em cores ou injeção de contraste de salina, sem verdadeira deficiência do SIA. O ‘‘FOP com fluxo da esquerda para a direita’’ ocorre quando a hemodinâmica atrial decorre da abertura potencial da comunicação do forame, resultando em passagem de sangue da esquerda para direita demonstrado pela imagem Doppler (Figura 7). Quando o FOP é estirado por hemodinâmica atrial, cria um defeito no septo, que é chamado de FOP ‘‘*stretched*’’, podendo resultar em fluxo de sangue da esquerda para a direita e/ou da direita para a esquerda, dependendo das diferenças nas pressões atriais.

## Aneurisma do Septo Atrial

**Figura 8:**
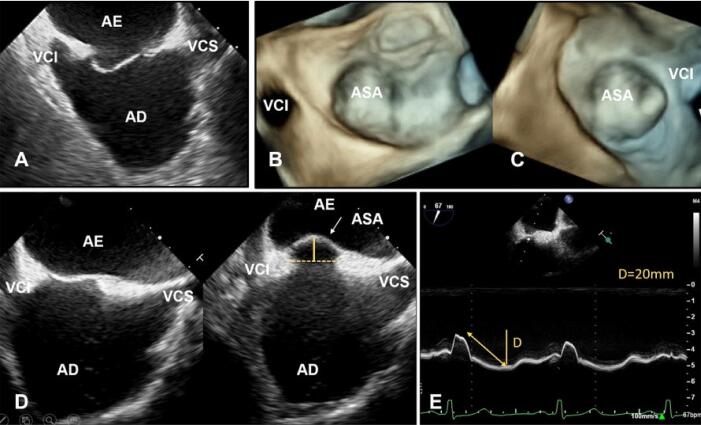
– Imagens ecocardiográficas transesofágicas do SIA. (A) Visão bidimensional bicaval mostrando deslocamento acentuado da lâmina da FO em direção ao AD, compatível com aneurisma. (B, C) Visão tridimensional en face (3D Zoom) do septo aneurismático de perspectiva atrial esquerda e direita, respectivamente. Imagens ecocardiográficas transesofágicas bidimensionais do septo interatrial. (D) Visão bicaval mostrando o deslocamento da lâmina da FO em direção ao AE, compatível com o ASA. (E) Modo M de ASA demonstrando mobilidade maior que 15 mm da lâmina da FO. AD: átrio direito; AE: átrio esquerdo; ASA: aneurisma do septo atrial; D: distância; FO: fossa oval; VCI: veia cava inferior; VCS: veia cava superior.

O Aneurisma do septo atrial (ASA) é uma redundância ou deformidade sacular do SIA, sempre limitado a lâmina da FO, e associado ao aumento da mobilidade do tecido septal. A melhor modalidade de imagem para visualizar o ASA é a ETE 3D (Figura 8).

O ASA é definido como uma excursão do tecido septal (lâmina da FO) ≥ 10 mm do plano septal para AD ou AE ou excursão total combinada direita-esquerda de 15 mm. A excursão do SIA pode ser documentada usando imagem 2D, bem como avaliação modo M quando o cursor modo M é alinhado perpendicularmente ao plano septal. Isso pode ser feito na incidência bicaval na ETE (Figura 8D, 8E).

A prevalência do ASA é de 2% a 3%^12^ e tem sido associado à presença e maiores tamanhos de FOP e prevalência aumentada de acidente vascular cerebral (AVC) criptogênico e outros eventos embólicos e múltiplas fenestrações septais, que devem ser avaliadas cuidadosamente usando imagem Doppler colorido. A presença e extensão do ASA é fator na seleção do dispositivo oclusor de FOP. Um dispositivo grande pode ser escolhido para abranger e estabilizar o SIA ou um menor para melhor conformação com o ASA.

## Válvula de Eustáquio e Rede Chiari

A Válvula de Eustáquio é o remanescente embriológico da válvula da VCI que, na vida fetal, direciona o fluxo da VCI através da FO, e se estende da junção entre a VCI e o AD. Considerada proeminente quando sua protrusão é ≥ 10 mm dentro do AD. A Válvula de Eustáquio grande ou proeminente no contexto de FOP, pode contribuir indiretamente para embolia paradoxal, impedindo o fechamento espontâneo do forame^13^ ou quando, próxima ao SIA, pode interferir na implantação do lado AD do dispositivo oclusor septal.

A Rede de Chiari é remanescente da válvula direita do seio venoso e aparece como malha de estruturas filamentosas em vários locais do AD, inclusive próximo a desembocadura da VCI e do seio coronário. Está presente em 2%-3% da população geral e está associada à presença de FOP e ASA.^14^ A Rede Chiari pode interferir na passagem de cateteres e dispositivo através do AD. Portanto, a identificação da presença de rede Chiari deve fazer parte da avaliação ecocardiográfica.

## Avaliação ecocardiográfica do septo interatrial

A ETE 2D oferece informações anatômicas incrementais comparado ao ecocardiograma transtorácico (ETT) para avaliação do SIA, também melhora a visualização do SIA e das estruturas adjacentes. O SIA tem estrutura anatômica tridimensional e dinâmica, não sendo verdadeiramente plano, com limitações em sua avaliação usando qualquer forma de ecocardiografia 2D. A imagem 3D oferece visões exclusivas do SIA, e, em particular, permite visão “*en face”* ou frontal da FO e seus arredores (Figura 2). A imagem biplanar bidimensional (ou triplanar) é uma modalidade advinda da tecnologia 3D, com vantagem de exibir e visualizar imagens ecocardiográficas simultâneas, com alta taxa de quadros e excelente resolução temporal (Figura 5A, 5B). Um plano ortogonal de imagem exibido simultaneamente fornece informações incrementais em comparação com a imagem uniplanar, e é adequada para orientação de procedimentos transcateter.^15^

ETE 2D x SIA: visões múltiplas e sequenciais devem ser usadas para avaliação completa e sistemática do SIA, determinando tamanho, forma e localização de qualquer CIA e sua relação com estruturas circundantes. Recomenda-se uma avaliação sequencial a partir das visões padrão com aumento gradual no ângulo do transdutor em séries de 15º de incrementos, para varrer o feixe de ultrassom através das áreas de interesse. O Doppler colorido é aplicado posteriormente com escala reduzida à aproximadamente 35-40 cm/s para identificar fluxos de baixa velocidade através de pequena fenestração, o FOP ou CIA menor. Na ETE, cinco visões de base são usadas para avaliar o SIA: Esôfago superior de eixo curto, esôfago médio de eixo curto da valva aórtica, esôfago médio quatro câmaras, esôfago médio bicaval e esôfago médio eixo longo.

ETE 3D x SIA: adquire-se dados volumétricos 3D em suas apresentações setorial limitada (*live* 3D), *zoom* e volume completo (*full-volume*) a partir de visões principais: esôfago médio de eixo curto, bicaval, bicaval sagital e quatro câmaras. Quando o SIA é visto a partir do AE, deve ser orientado com a veia pulmonar superior direita na posição de 1 hora. Quando exibido do AD, a VCS deve estar localizada na posição de 11 horas (Figura 2D, 2E).

## Avaliação ecocardiográfica do Forame Oval Patente

O ETT é usado para a avaliação inicial de FOP; entretanto, a ETE é necessária para avaliação mais abrangente de anormalidades septais, por melhor qualidade da imagem. A ETE não é invariavelmente necessária para avaliação de FOP se o fechamento não estiver sendo considerado, porém deve ser realizado em todos os pacientes avaliados para procedimento percutâneo ou cirúrgico.

**Figura 9:**
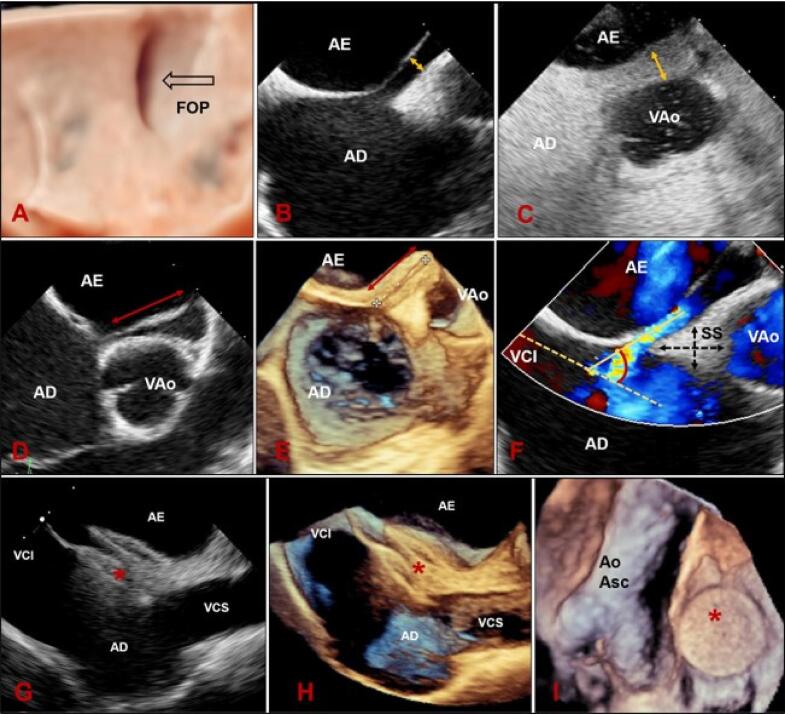
– (A) Visão ETE 3D (TrueVue) en face do orifício elíptico do FOP. (B, C) visão ETE 2D mostrando aumento da altura do túnel do FOP após manobra de Valsalva. (D, E) visão ETE 2D e 3D, respectivamente, com ajustes no plano de imagem para medida do túnel do FOP. (F) visão ETE 2D, ajustada para medida do ângulo VCI-FOP e medida da espessura e extensão do SS. Imagens ecocardiográficas transesofágicas durante procedimento de oclusão percutânea de FOP. (G) Visão bicaval mostrando dispositivo oclusor (*) bem posicionado, com discos planos e tecido septal entre os discos. (H) Imagem bicaval 3D confirmando posicionamento adequado do dispositivo. (I) Visão en face de perspectiva atrial direita mostrando o dispositivo oclusor e estruturas adjacentes. AD: átrio direito; AE: átrio esquerdo; Ao Asc: aorta ascendente; FOP: forame oval patente; SS: septo secundum; VAo: valva aórtica; VCI:veia cava inferior; VCS: veia cava superior.

As visões com ETE usadas para a avaliação de FOP começam no plano do esôfago médio com configurações otimizadas para visualizar o SIA, e o plano de imagem ecocardiográfico deve ser girado, começando em ângulos multiplanares de 0º, com incrementos de 15º, para avaliação septal completa. Imagens lado a lado com Doppler colorido em escala baixa (35-40 cm/s) são úteis para identificar fluxo através do FOP e possíveis defeitos septais adicionais. A sonda poderá ser levemente retirada para melhor avaliação do SIA próximo à VCS e aprofundada para melhor avaliação do SIA próximo à VCI. A partir de 30º-50º, com a VAO em corte transversal, o FOP deve ser visualizado adjacente à aorta (Figura 7D, 7G, 7H). A rotação do plano de imagem deve alinhar na imagem o túnel do FOP. Deste ângulo, o comprimento do túnel pode ser avaliado, assim como medida a espessura do SS^15^ (Figura 9A-9F).

Com o FOP visualizado, contraste salino é injetado para avaliar *shunt* direita-esquerda. Detalhes anatômicos importantes do SIA incluem localização do FOP (região anterior ou superior da FO), comprimento total do SIA, espessura e extensão do SS, comprimento do túnel do FOP, tamanho do FOP, ângulo entre a VCI e FOP, presença de ASA e fenestrações ou defeitos do SIA (Figura 9A, 9B, 9C).

A ETE 3D é usada para definir melhor as variações do FOP e mostrar que sua forma é elíptica (Figura 9A). A área do FOP muda durante o ciclo cardíaco e é maior durante a sístole ventricular. A ETE 3D também é usada para guiar o procedimento de fechamento com visões “*en face”* do SIA, mostrando a relação do FOP e do dispositivo com estruturas adjacentes (Figura 9G-9I).

Características anatômicas específicas do FOP devem ser avaliadas ao decidir sobre a seleção do dispositivo oclusor. Especificamente o comprimento do túnel do FOP, presença e tamanho de ASA, espessura do SS e tamanho máximo do FOP durante o ciclo cardíaco são importantes na seleção apropriada do dispositivo e incidência menor de complicações em comparação com a estratégia de dispositivo único para todo FOP.^16^

## Caracterização do Shunting

O Doppler colorido pode detectar o *shunt* em FOP; contudo, o *shunt* é frequentemente intermitente e pode não ser facilmente detectável. Quando um FOP é estirado (*stretched*) por diferenças na pressão no AE e AD, o *shunt* da esquerda para a direita pode ser visto no Doppler colorido (Figura 7). A ecocardiografia com contraste usando macrobolhas de solução salina agitada combinada com manobras fisiológicas, aumenta a sensibilidade da detecção de FOP.^17,18^ Macrobolhas geradas com agitação são grandes para passar a vasculatura pulmonar normal e são facilmente detectadas por imagem ecocardiográfica por sua ecogenicidade aumentada (Figura 7H). O ETT com contraste pode ser usado para detectar o FOP com sensibilidade e especificidade razoáveis; entretanto, a ETE é considerada o padrão de referência. Seja usando ETT ou ETE, a precisão do teste será melhorada pelo uso de protocolo padronizado, como abaixo, que inclui múltiplas injeções de solução salina e manobras provocativas para aumentar a pressão do AD, como a manobra de Valsalva (MV), tosse ou compressão abdominal.^19,20^

Protocolo (Figura suplementar 1):

- Inserir cateter intravenoso na veia antecubital, conectado a torneira de três vias.
- Combinar em seringa de 10 mL conectada à torneira, 8 mL de solução salina mais 1 mL de sangue do paciente, mais 1 mL de ar; adição de sangue a solução de contraste resulta em aumento da intensidade das MB detectadas pela ecocardiografia.^20^
- Agitar a solução rapidamente para frente e para trás entre duas seringas de 10 mL anexada a torneira de três vias para produzir bolhas.
- Injetar a solução agitada rapidamente na veia antecubital junto com aquisição ecocardiográfica de clipe longo de imagem (cerca de 10 batimentos), registradas a partir da visão quatro câmaras no ETT, e a melhor visão do SIA na ETE, geralmente está entre 30º-100º.
- Uso de imagens biplanares pode melhorar a detecção de pequenos fluxos de sangue da direita para a esquerda.

Aparecimento de macrobolhas no AE dentro de 3-6 batimentos cardíacos após a opacificação do AD é considerada positiva para presença de *shunt* intracardíaco. Idealmente, as bolhas serão visualizadas cruzando o SIA através do FOP (10E, 10G).

Manobras fisiológicas que aumentem transitoriamente a pressão do AD são necessárias para promover uma passagem da direita para a esquerda das macrobolhas, a fim de identificar o FOP quando nenhum *shunt* estiver presente. A MV é comumente realizada e deve ser mantida por tempo suficiente para que as bolhas preencham o AD, e sua eficácia pode ser avaliada via ecocardiografia pela presença de abaulamento para a esquerda do SIA, indicando obtenção de pressão no AD maior que AE. O aparecimento de macrobolhas no AE após 3-6 batimentos cardíacos indica *shunt* intrapulmonar, como malformação arteriovenosa, que é confirmada quando as bolhas são visualizadas entrando no AE a partir das veias pulmonares e não visualizadas cruzando o SIA (11A-11D). Outras razões para estudo falso-positivo de bolhas para FOP são defeito do septo seio venoso ou outra CIA não identificada.

Estudos com bolhas podem resultar em achados falso-negativos devido à opacificação inadequada do AD, a MV inadequada, presença de válvula de Eustáquio direcionando o retorno venoso da VCI para o SIA (impedindo que MB oriundas da VCS cruzem o septo), incapacidade de aumentar a pressão do AD acima da pressão do AE, como na disfunção diastólica ventricular esquerda, e baixa qualidade de imagem. Com essa qualidade, o uso de imagens de segunda harmônica pode melhorar a identificação e detecção de macrobolhas. Uma injeção na veia da perna também pode, raramente, ser usada para superar a rede de Chiari ou válvula de Eustáquio muito grandes que acabam impedindo as bolhas de preencherem o AD a partir da VCS. Pode-se quantificar o *shunt* da direita para a esquerda com base no número de bolhas que aparecem no coração esquerdo em quadro ecocardiográfico estático; entretanto, este número depende da quantidade de macrobolhas injetadas e de uma MV adequada.

## Ecocardiografia no fechamento transcateter

Uma imagem ecocardiográfica é usada para auxiliar fechamento percutâneo de FOP, fornecendo informações significativas na seleção do paciente e dispositivo, orientação do procedimento, monitoramento de complicações e avaliação dos resultados. A escolha do dispositivo oclusor deve levar em conta anatomia do SIA e do FOP, disponibilidade do dispositivo e experiência da equipe. A escolha habitualmente privilegia dispositivos próprios para fechamento dessa estrutura, entretanto, em casos específicos, em razão da análise da anatomia, o dispositivo escolhido pode ser um que tenha sido idealizado para oclusão de CIA.^21^[Fig f11]

**Figura 10 f11:**
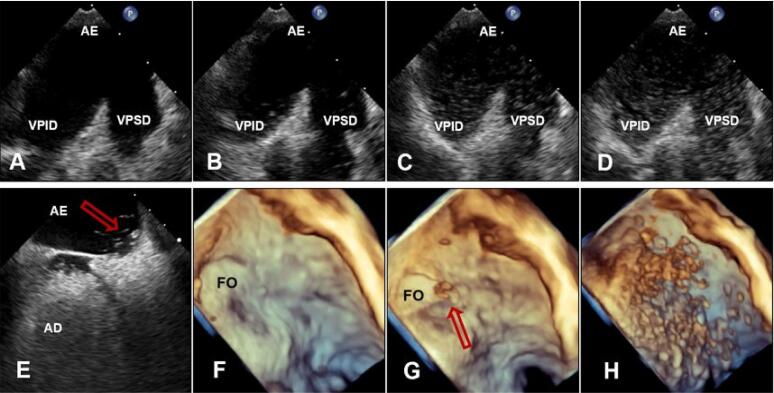
– Imagens ecocardiográficas transesofágicas. (A, B, C, D) mostram visões bidimensionais de microbolhas de salina entrando no AE a partir das veias pulmonares direitas em paciente com fístula arteriovenosa pulmonar. (E) visão bidimensional bicaval mostrando microbolhas cruzando o septo atrial através da FO, confirmando presença de FOP. (F, G, H) Visões tridimensionais en face de perspectiva atrial esquerda, confirmando a chegada das bolhas no AE (seta) através do FOP. AD: átrio direito; AE: átrio esquerdo; FO: fossa oval; FOP: forame oval patente; VPID: veia pulmonar inferior direita; VPSD: veia pulmonar superior direita.

Em outubro de 2016, o *Food and Drug Administration* dos EUA (FDA) aprovou uso do oclusor Amplatzer PFO (St. Jude Medical, Inc.) para fechamento percutâneo de FOP e tornou-se o primeiro dispositivo comercialmente disponível nos Estados Unidos para uso em pacientes com AVC criptogênico presumidamente mediado por FOP. Três dispositivos estudados no Estados Unidos em grandes ensaios clínicos randomizados são: Oclusor Amplatzer PFO, oclusor septal StarFlex (NMT Medical, Inc.) e o Helex/Cardioform septal oclusor (Gore & Associates).

## Embolização e erosão por dispositivo

Complicações dos dispositivos oclusores do FOP são raras e incluem embolização do dispositivo, perfuração cardíaca, tamponamento e erosão.^22^

A embolização do dispositivo ocorre em aproximadamente 0,1%–0,4% dos casos, e é mais comum com dispositivos de fechamento de CIA.^22^ É potencialmente fatal e requer remoção imediata por intervenção percutânea ou cirúrgica, sendo diagnosticada por ETT de rotina, que avalia a localização do dispositivo deslocado e sequelas fisiológicas (obstrução de entrada/saída, ruptura valvar) que resultam da embolização.

A erosão por dispositivo é rara, mas potencialmente fatal, podendo ocorrer com vários dispositivos, e atinge o teto do AD ou AE ou junção da aorta e pode resultar em hemopericárdio, tamponamento, fístula aórtica e/ou morte.^23^ A erosão pode começar como um evento subclínico, com o dispositivo colidindo com estruturas circundantes, tensionando tecido atrial ou aórtico, ou resultando em derrame pericárdico subclínico. A maioria das erosões ocorre na primeira semana após a implantação.^24^ Os fatores mais significativos para esta complicação são o superdimensionamento do dispositivo (40% dos casos), ausência completa de borda aórtica, localização septal alta/superior do defeito e insuficiência associada da borda posterior. Importantes fatores de risco para erosão após a colocação do dispositivo incluem deformação do dispositivo de fechamento na raiz aórtica e derrame pericárdico observado dentro de 24 horas de implantação.

## Imagem do SIA imediatamente após o procedimento

Uma avaliação ecocardiográfica é realizada para confirmar que os discos direito e esquerdo do dispositivo oclusor estejam planos e apostos ao septo com tecido septal entre os discos. Uma avaliação completa do dispositivo, do SIA e das estruturas adjacentes é realizada antes da liberação (Figura 9G-9I). Uma avaliação de Doppler colorido é realizada para excluir fluxo residual nas margens do dispositivo, cuja presença sugere tamanho ou posição inadequados do dispositivo. Interferência com veias pulmonares, seio coronário, função das valvas atrioventriculares e deformação da raiz da aorta são cuidadosamente avaliadas e excluídas antes da liberação.

## Forame Oval Patente e acidente vascular cerebral criptogênico

O AVC criptogênico é definido como infarto cerebral não atribuível a fonte definida de cardioembolismo, aterosclerose de grandes artérias, doença de pequenas artérias ou outras etiologias determinadas, tais como: vasculopatias não ateroscleróticas, estados de hipercoagulabilidade ou distúrbios hematológicos, apesar de extensiva avaliação vascular, cardíaca e sorológica – critério *TOAST (Trial of Org 10172 in Acute Stroke Treatment)*.^25^ Afeta cerca de 25% a 40% dos pacientes com isquemia cerebrovascular.^26^

O AVC embólico de origem desconhecida é uma subcategoria de AVC criptogênico isquêmico que denota especificamente AVC não-lacunar sem etiologia imediatamente identificável.^27^

O FOP tem sido associado a múltiplas patologias: enxaqueca; síndrome platipnéia ortodeoxia, doença de descompressão de alta altitude e relacionada ao mergulho; e mais comumente, o AVC criptogênico.^28-30^

A associação entre AVC criptogênico e FOP foi creditada pela primeira vez ao patologista Julius Cohnheim em 1877. Entretanto, o interesse moderno começou após Lechat et al., em 1988 observarem que o FOP estava presente em 40% dos indivíduos com AVC criptogênico em comparação com apenas 10% em controles.^31^ Desde então, outros estudos documentaram a associação de FOP com pacientes que sofrem AVC, particularmente em idade < 55 anos.^32^

O FOP tem sido associado ao AVC criptogênico por embolia paradoxal, na qual coágulo ou partícula embólica da circulação venosa periférica passa para circulação arterial através de um *shunt* direito-esquerda, contornando o sistema de filtração natural na vasculatura pulmonar, ou ainda, trombose *in situ* no túnel do FOP, estase sanguínea local no lado esquerdo do SIA, ou formação de trombo dentro do ASA concomitante ao FOP, e, indiretamente, através do aumento da suscetibilidade a arritmias. Há indicações de que a atividade elétrica do AE pode ser alterada na presença do FOP, o que aumenta ainda mais o risco de AVC. O estiramento do SIA induzido por aberrações septais (túnel grande do FOP, ASA) podem modificar a despolarização atrial, ativando substrato arritmogênico.^33^ Trombos atravessando o FOP podem ser eventualmente observados tanto em autópsias quanto em alguns poucos relatos de exames ecocardiográficos (Figura suplementar 2), sugerindo este mecanismo como um possível responsável de embolia paradoxal (Figura Central).

Apesar de pouco frequente visualização de trombos em FOP, a observação epidemiológica indica que ele é responsável por um número considerável de AVCs. Históricos de trombose venosa profunda, embolia pulmonar, hipertensão arterial pulmonar, viagens prolongadas, MV precedendo início de sintomas de AVC, enxaqueca e apneia do sono têm sido descritos como fatores de risco independentes para associação entre o FOP e o AVC.^34^ Outros mecanismos podem ser responsáveis por AVC criptogênico, tais como a fibrilação atrial (FA) não detectada ou oculta. A FA oculta está associada ao aumento de 2,5 vezes no risco de AVC, embora com baixa relação temporal.^35^ Assim também, valvopatia mitral, contraste espontâneo em AE, calcificação do anel mitral, próteses cardíacas, tumores cardíacos, ateroma aórtico e distúrbios hematológicos com suscetibilidade à formação de trombos.^36^ É proposta propedêutica mínima para AVC de origem indeterminada com tomografia/ressonância de crânio, ecocardiograma, imagem extra/intravascular, pesquisa de distúrbios hipercoaguláveis e monitorização cardíaca por ≥ 24 horas.^37,38^ A monitorização cardíaca é importante para desmascarar arritmias ocultas, especificamente, a FA que ainda é a causa mais comum de AVC criptogênico.

O ETT e, especialmente, a ETE com utilização de bolhas de salina, faz parte da avaliação do FOP, desde a detecção do *shunt*, características anatômicas do FOP, diagnóstico diferencial com outras comunicações atriais ou *shunt* pulmonar.^39^

O Doppler transcraniano é mais sensível, porém menos específico que a ETE no diagnóstico de FOP. Sua insensibilidade de fazer diagnóstico diferencial entre *shunt* cardíaco e pulmonar e impossibilidade diagnóstica das alterações anatômicas do FOP justificam a menor especificidade.^40^

Entretanto, há controvérsia sobre manejo ideal do FOP no cenário de AVC criptogênico. A base do tratamento era terapia antitrombótica, derivada dos estudos FRENCH PFO-ASA^41^ e PICSS.^42^ Estes não mostraram risco aumentado de recorrência de AVC independentemente da presença de FOP. Apesar da hipótese embólica do AVC criptogênico no cenário de FOP, a anticoagulação não foi superior à aspirina na prevenção de AVCs recorrentes. Estes achados também foram replicados no estudo NAVIGATE ESUS^43^ que encontrou redução não estatisticamente significativa de AVC isquêmico com anticoagulação, se comparado à aspirina. Entretanto, no AVC criptogênico, a taxa de recorrência relatada é substancial, e pode variar de 3% a 6%.^37^ Portanto, é inevitável considerar opções de tratamento mecânico para FOP. O fechamento cirúrgico do defeito trouxe morbidade significativa e sem evidência de benefício na prevenção de recorrências.^44^

## Evidência para fechamento percutâneo do FOP

O fechamento percutâneo do FOP está associado a significativamente menos morbidade do que cirurgia, e é opção na embolia paradoxal. Na última década, o desafio foi demonstrar o benefício na população com baixa incidência de AVC. Dois ensaios principais, CLOSURE I^45^ e PC trial^46^ não conseguiram demonstrar clara superioridade do fechamento percutâneo, em relação à terapia medicamentosa. Houve, entretanto, um aumento na incidência de arritmias atriais no grupo FOP de fechamento por dispositivo. Em seguida, o estudo RESPECT^47^ mostrou que fechamento do FOP com Amplatzer PFO Occluder foi superior ao manejo medicamentoso na redução de AVC isquêmico recorrente a longo prazo em pacientes com AVC criptogênico e evidência de FOP. Com base nos dados cumulativos dos estudos, as diretrizes do AHA/ASA 2014 forneceram recomendação Classe III para fechamento do FOP em pacientes com AVC criptogênico e nenhuma evidência de trombose venosa profunda.^48^

Entretanto, ensaios iniciais tiveram limitações significativas. Eles utilizaram dispositivos mais antigos associados a maiores taxas de complicações e tempo de acompanhamento com uma média apenas de 2-4 anos. Resultados favoráveis a longo prazo foram relatados por ensaios observacionais.^49,50^ Além disso, metanálises de pacientes demonstraram superioridade do uso do Dispositivo Oclusor Amplatzer PFO.^51,52^

Posteriormente, três ensaios clínicos randomizados adicionais, CLOSE,^53^ DEFENSE-PFO^54^ e REDUCE^55^ mostraram superioridade do fechamento percutâneo do FOP, em comparação com a terapia medicamentosa isolada.

Múltiplas metanálises avaliaram evidências de fechamento do FOP. Nasir et al., investigaram os seis principais ensaios avaliando fechamento do FOP: CLOSURE I, PC trial, RESPECT, CLOSE, REDUCE e DEFENSE-PFO com um total de 3.510 pacientes. Esse estudo mostrou o benefício persistente do fechamento do FOP versus antiplaquetário ou anticoagulação isolada, com um número necessário para tratar de 41 (OR 0,34, IC 95% 0,15–0,79, p = 0,012). Efeitos colaterais principais foram comparáveis, sem diferença na mortalidade ou eventos hemorrágicos maiores.^56^ Wintzer-Wehekind et al., demonstraram que, com fechamento percutâneo do FOP, e após a descontinuação da terapia antiplaquetária, não houve aumento de eventos no seguimento de longo prazo.^57^ (Tabela suplementar 1) mostra os principais estudos randomizados neste cenário.

Vários estudos também buscaram determinar, com maior precisão, quais pacientes mais se beneficiariam do tratamento intervencionista. Dados de estudos recentes sugerem que, associado ao ASA, o FOP de maior tamanho e septo hipermóvel pode obter maior benefício do fechamento. Em todos os ensaios discutidos, a ETE foi usada como modalidade de imagem para avaliar o FOP. Enquanto não havia valores de corte recomendados para uso nesses ensaios, CLOSE incluiu apenas o FOP com > 30 bolhas (MB) ou ASA associado > 10 mm, e REDUCE estratificou o tamanho do *shunt* em quatro grupos (0 MB sem *shunt*; 1–5 MB pequeno; 6–25 MB moderado; > 25 MB grande) 81% com *shunts* moderados ou grandes.

Apesar dos resultados positivos dos dados de fechamento de FOP, há limitações. Em cinco dos seis estudos, o limite de idade superior foi de 60, com o restante sendo insuficiente para maiores de 60 anos. Portanto, os resultados não podem ser generalizados para toda população.^56^ Houve um aumento de 4,7% no risco de nova FA no grupo de fechamento em comparação ao grupo tratamento medicamentoso.^58^ Isso pode ser atribuído a irritação atrial durante o procedimento, pois a maioria dos eventos foi periprocedimento e transitório. Adicionalmente, existem complicações raras do procedimento, incluindo perfuração atrial com tamponamento exigindo remoção cirúrgica do dispositivo. Por fim, está o risco potencial de longo prazo de dilatação radicular e subsequentes erosões causadas pelo dispositivo,^59^ bem como potencial formação de trombos no dispositivo.^60^

## Candidatos para fechamento Do FOP

Apesar das evidências que ligam FOP e AVC criptogênico, nem todo FOP precisa ser fechado. Cerca de um terço dos FOPs são encontrados acidentalmente, com outras etiologias mais plausíveis de AVC.^50^ Se a investigação convencional não revelar a causa do AVC, a triagem do paciente para fechamento percutâneo do FOP, de acordo com fatores de risco específicos do paciente, está justificada.

Para ajudar a identificar pacientes com AVC e FOP nos quais o FOP é a causa provável, o Escore de Risco de Embolia Paradoxal (RoPE) foi desenvolvido. (Conforme a Tabela suplementar 2)**.**

Esse índice foi criado usando dados de regressão de 12 bancos de dados envolvendo pacientes com AVC criptogênico^61,62^ e leva em consideração dados clínicos como histórico de hipertensão, diabetes, AVC ou acidente isquêmico transitório (AIT), tabagismo, infarto cortical evidenciado em exames de imagem e idade do paciente. Quanto maior a pontuação, maior a probabilidade do FOP ser a causa do AVC criptogênico (Tabela suplementar 3).

As pontuações 7, 8 e 9-10 correspondem, respectivamente, a 72%, 84% e 88% de chance de que o FOP seja a causa do AVC criptogênico e define um subconjunto de pacientes que pode se beneficiar do fechamento. Além disso, o baixo escore RoPE ≤ 6 foi identificado como fator de risco independente para mortalidade e AVC isquêmico recorrente após fechamento do FOP, juntamente com outros fatores de risco, como maiores dimensões do átrio esquerdo.^63^ Pacientes jovens, com AVC superficial e sem fatores de risco vascular, apresentam escore elevado. Em pacientes com escore baixo, idosos com fatores de risco vasculares, a presença de FOP sugere ser acidental. O risco de AVC ou AIT é calculado em 2 anos para cada grupo.

Um estudo recente observacional de 107 pacientes com e sem AVC criptogênico que foram programados para fechamento percutâneo de FOP, tiveram as características anatômicas dele avaliadas por ETE e demonstraram que algumas características anatômicas do FOP predispõem a formação e passagem de trombos do AD para o AE sendo a causa de embolias sistêmica.^64^

As características ecocardiográficas do FOP avaliadas foram:

- **Altura do FOP:** separação máxima entre SP e SS no frame sistólico final. Considerada grande quando ≥ 2,0 mm.
- **Comprimento do Túnel:** sobreposição máxima entre SP e SS. Comprimento ≥ 10 mm foi definido como FOP de túnel longo, que pode ser um espaço para a formação de trombos.
- **Grau do *Shunt* Direita-Esquerda:** avaliado em repouso e após MV, utilizando-se contraste de salina. Número de MB é contado em único frame, e > 20 MB é considerado grande *shunt*, com maior risco de AVC.
- **Ângulo entre VCI e *Flap* do FOP:** medido em plano de imagem que exibe a VCI e o SIA. Definido baixo ângulo de FOP quando ≤ 10º podendo direcionar preferencialmente o fluxo sanguíneo da VCI para o SIA e o orifício do FOP, sendo considerado de maior risco para AVC.
- **Aneurisma do Septo Interatrial:** tecido redundante, móvel, na região da FO, com excursão septal fásica da linha média para AD ou AE ≥ 10 mm ou excursão total ≥ 15 mm entre AD e AE.
- **Hipermobilidade do Septo Interatrial:** septo móvel com excursão ≥ 5 mm em cada batimento cardíaco. Mecanismos que têm sido propostos como responsáveis por embolia paradoxal causada por aneurisma ou hipermobilidade do SIA são:

1. Embolia paradoxal por passagem de trombo do AD para AE através do FOP.
2. ASA sem *shunt* intracardíaco, pequenos trombos de fibrina e plaquetas podem se formar no lado esquerdo do septo, desprendendo-se com oscilação do aneurisma.

- **Válvula de Eustáquio e Rede de Chiari:** podem direcionar o fluxo, que chega pela VCI diretamente para o SIA, favorecendo o FOP e ASA, e, indiretamente, facilitando embolia paradoxal. O FOP com grande *shunt* direita-esquerda foi observado com maior frequência nos pacientes que tinham rede de Chiari.^65^

Características ecocardiográficas que aumentam o risco de AVC incluem grande tamanho do FOP, grande *shunt* direita-esquerda, *shunt* espontâneo direita-esquerda, maior mobilidade da lâmina do FOP, válvula de Eustáquio proeminente, rede de Chiari e ASA. A análise multivariada dessas observações evidenciou fatores ecocardiográficos que foram preditores independentes de eventos cerebrais isquêmicos demonstrados na Tabela suplementar 4.

Baseado nesses dados anatômicos analisados pela ecocardiografia, foi elaborado um escore de risco para o FOP, sendo responsável pela embolia paradoxal em pacientes que apresentarem AVC (Tabela suplementar 5).

Escores elevados são observados em pacientes jovens com AVC superficial sem ou com poucos fatores de risco tradicionais. Pacientes com escores baixos, com fatores de risco vascular e idosos sugerem que o FOP é acidental e não diretamente responsável pelo evento isquêmico. O risco de AVC/AIT é calculado para um período de 2 anos.

Essas observações são apoiadas por achados dos ensaios CLOSE e DEFENSE-PFO. Estudos que incluíram apenas o FOP de alto risco, demonstrando ausência de AVC recorrente no braço fechamento aos 5 e 2 anos, respectivamente. Em contraste, pacientes com AVC isquêmico também foram relatados como tendo FOP de túnel curto.^66^ Assim, mais estudos são necessários para elucidar a relação entre comprimento do túnel de FOP e AVC, identificando mecanismos.

Determinação da gravidade do *shunt* pelo número de bolhas que cruzam o SIA através do FOP pode não ser completamente precisa, pois algumas bolhas podem não estar no plano de imagem. É provável que haja pouca diferença entre o *shunt* com 20 e 21 bolhas, mas nesta análise, um é classificado como pequeno e o outro como grande. Além disso, a determinação dicotômica da gravidade do *shunt* (pequeno vs grande) pode ser propensa a viés significativo. A MV depende substancialmente do esforço do paciente, e essa não uniformidade pode afetar as características morfométricas do FOP.

## Conclusões

Nos últimos anos, o influxo de evidências mostrando o benefício do fechamento percutâneo do FOP no cenário de AVC criptogênico, em comparação com terapia medicamentosa isolada e a evolução dos procedimentos percutâneos envolvendo cardiopatia estrutural e procedimentos eletrofisiológicos, trouxe interesse adicional na anatomia do SIA. Com base nos dados atuais, além de avaliação completa do paciente, uma caracterização anatômica detalhada e padronizada do SIA são recomendados.
