# Supplementary material for: Papel do Ecocardiograma na Avaliação do Septo Interatrial e Pesquisa de Forame Oval Patente como Fonte Emboligênica
Source: Arq Bras Cardiol. 2023 Oct 16;120(9):e20220903. [Article in Portuguese] doi: 10.36660/abc.20220903 (PMC10586815; doi:10.36660/abc.20220903)
Supplement: Translation

# Translation: The Role of Echocardiography in the Assessment of the Interatrial Septum and Patent Foramen Ovale as an Emboligenic Source

**Authors:** Angele Azevedo Alves Mattoso, Joberto Pinheiro Sena, Viviane Tiemi Hotta

**Affiliations:** 1 Brazil Hospital Santa Izabel, Salvador, BA – Brazil; 2 Brazil Hospital Santa Izabel – Hemodinâmica, Salvador, BA – Brazil; 3 Brazil Instituto do Coração HC-FMUSP – Unidade Clinica de Miocardiopatias e Doenças da Aorta, São Paulo, SP – Brazil; 4 Brazil Fleury Medicina e Saúde, São Paulo, SP – Brazil

**Keywords:** Echocardiography, Septo Atrial, Foramen Ovale, Patent

## Abstract

Atrial septal defects (ASD) account for approximately 6%-10% of congenital heart defects, with an incidence of 1 in 1,500 live births.^1^ Patent foramen ovale (PFO) is more common and is present in more than 20%-25% of adults.^2^ Clinical syndromes associated with ASD and PFO are variable, and their implications are targeted by pediatric and adult medicine, neurology, and surgery.

Additional interest in the anatomy of the interatrial septum (IAS) has increased substantially over the last two decades. Additionally, percutaneous procedures involving left-sided structural heart disease and electrophysiological procedures have evolved considerably. Ideally, these catheter-based interventions require a direct route to the left atrium (LA) through the IAS, with a full understanding of its anatomy. Also, sophisticated and noninvasive imaging technologies such as two-dimensional transesophageal echocardiography (2D-TEE) and three-dimensional transesophageal echocardiography (3D-TEE), cardiac magnetic resonance imaging (CMR), and computed tomography (CT) have evolved considerably, providing anatomical details of cardiac structures visualized in 2D and 3D format and being key for the diagnosis and treatment of patients with heart diseases.

Therefore, assessing the anatomy of the IAS and any abnormalities requires a standardized and systematic approach, integrating diagnostic modalities and enabling adequate and consistent evaluation for both surgical and transcatheter therapies.

## Anatomy of the interatrial septum

The IAS has three components: septum primum (SP), septum secundum (SS), and atrioventricular canal septum. The sinus venosus is not a component of the true IAS but is an adjacent structure through which atrial communication (defect) can occur.^3,4^

The atria first develop as a common cavity. At approximately 28 days of gestation, the SP, derived from the atrial roof, begins to migrate toward the developing endocardial cushions. During this transition, the space between the SP and the endocardial cushion is called the “embryonic ostium primum” or “foramen primum” (Figure 1A, 1B).

**Figure 1:** – Scheme demonstrating the normal development of the IAS. (A) The SP grows from the atrial roof. (B) Fenestrations develop within the SP. (C) The SS develops by a folding of the atrial wall. The ostium secundum acts as a conduit for the right-to-left passage of oxygenated blood. (D) The SP and SS remain unfused at the anterosuperior edge of the FO, constituting the PFO. The arrow shows blood flowing through the PFO from the embryonic RA to the LA. The blue and purple dots represent the development of inflow from the vena cava and pulmonary to the atria. EC: endocardial cushion; FO: fossa ovalis; PFO: patent foramen ovale; OP: ostium primum; OS: ostium secundum; IAS: interatrial septum; SP: septum primum; SS: septum secundum.

The SS develops to the right of the SP, extending superiorly, posteriorly, and inferiorly and is the result of an infolding of the atrial roof rather than a true membranous structure (Figure 1C); SP overlapping on the left atrial side becomes the floor of an oval depression (fossa ovalis), delimited by the resulting edge of the SS infolding, seen on the right atrial side. The ostium primum closes by the fusion of the mesenchymal cells of the SP with endocardial cushions. At two months of gestation, the SS and SP fuse, leaving the foramen ovale as the only residual communication (Figure 1D). The SP acts as a flap valve in fetal life, directing blood flow from the right atrium (RA) to the LA through the ostium secundum or foramen ovale, which is essential to supply oxygenated blood from the placenta to vital organs. After birth, this opening closes when higher pressure in the LA pushes the valve flap against the muscular rim. If adhesion is incomplete around the entire margin of the rim, the PFO remains (Figure 2A, 2B, 2C).

**Figure 2:** – Anatomical specimen of the IAS from right atrial (A, B) and left atrial (C) perspective showing the SP and SS not completely fused, which constitutes the PFO. The arrow shows the port where blood flows through the PFO from the RA to the LA, courtesy of Dr. Vera Demarchi Aiello, Pathological Anatomy Laboratory of the Heart Institute of the Hospital das Clínicas of FMUSP. Three-dimensional transesophageal echocardiographic images (3D Zoom mode) of the IAS and adjacent structures. En face or frontal views (3D Zoom mode) of the IAS from right atrial (D) and left atrial (E) perspective, respectively. (F, G) En face views (3D Zoom mode – TrueVue) of the IAS showing the FO floor (arrow). Asc Ao: ascending aorta; FO: fossa ovalis; PFO: patent foramen ovale; CS: coronary sinus; NC: non-coronary aortic sinus; SP: septum primum; SS: septum secundum; IVC: inferior vena cava; SVC: superior vena cava; MV: mitral valve; RUPV: right upper pulmonary vein.

When observed from a right atrial perspective, the IAS appears as an extensive area, delimited inferiorly and superiorly by the orifices of the inferior vena cava (IVC) and superior vena cava (SVC), respectively; anteroinferiorly, this surface is limited by the orifice of the coronary sinus, anterosuperiorly by the non-coronary aortic sinus and, finally, anteriorly by the implantation region of the tricuspid valve leaflet. The 3D-TEE is the best imaging modality to describe this surface and its anatomical relationships from left and right atrial perspectives with accuracy comparable to anatomical specimens (Figure 2D, 2E, 2F, 2G). Anatomists describe the IAS as an interposed separation between the RA and the LA. Therefore, IAS puncture creates a direct communication between the atria without invading epicardial tissues. However, this definition actually corresponds to the floor of the fossa ovalis (FO), which resembles a crater-type depression when the right atrial surface of the septum is visualized “*en face* or frontally” (Figures 2A, 2B, 2D, and 2F) and as a superimposed thin wall on the left atrial side (Figures 2C, 2E, and 2G).

The FO is, therefore, the embryonic SP, covering about 20% of the aforementioned area, defined as the “true septum.”^4^ The muscle area that surrounds the superior, posterior, and inferior margins of the FO, which at first appears to be interposed between the atria, the “septum secundum or limbus of the FO,” as we have seen, does not actually separate the RA from the LA. Dissection of this area shows that it is an infolding of the atrial wall, and the puncture of this internal region of the RA produces an outflow from the atrial cavities to the “external” adipose tissues that occupy the fold. Thus, this infolding is undoubtedly a “false septum” (Figure 3).

**Figure 3:** – (A) Anatomical specimen showing the muscular area that surrounds the FO, interposed between the RA and the LA, the “SS” or “FO limbus,” which is an infolding of the atrial wall. Puncture of this internal region of the RA produces an outflow from the atrial cavities to the “external” adipose tissues that occupy the fold (“false septum”). (B) Cross-sectional CMR image obtained in a patient with lipomatous hypertrophy of the IAS, showing two “pseudomasses” filled with adipose tissue located superiorly, adjacent to the superior vena cava and inferiorly to the atrioventricular septum. RA: right atrium; LA: left atrium; FO: fossa ovalis; SS: septum secundum.

However, atrial wall infolding is referred to as the SS for consistency with the literature. Correctly described in the 19th century by Waterston^5^ and known as Sondergaard’s interatrial groove, this infolding is filled with a variable amount of epicardial adipose tissue and small vessels. The 2D-TEE can potentially identify adipose tissue with a characteristic hourglass appearance. However, the interatrial groove is almost virtual in normal individuals. Consequently, it is difficult to distinguish a small amount of adipose tissue between the two atrial walls that resemble a single wall. In other patients, the adipose tissue is particularly intrusive, invading the interatrial groove and separating the atrial walls (Figure 4).

**Figure 4:** – Transesophageal echocardiographic images of the IAS through the bicaval view showing different FO sizes. (A, F) Presence of significant adipose tissue in the SS or interatrial groove (arrows), giving the IAS an hourglass appearance. (B, D, E) Little adipose tissue, making the atrial groove (arrow) almost virtual. (C) Significant accumulation of adipose tissue in the infolding of the septum (invading the interatrial groove or SS) (red arrows), without compromising the FO lamina (white arrows), the so-called lipomatous hypertrophy. RA: right atrium; LA: left atrium; FO: fossa ovalis; IAS: interatrial septum; SS: septum secundum; IVC: inferior vena cava; SVC: superior vena cava.

CT can identify adipose tissue based on attenuation thresholds. However, despite the high spatial resolution of the technique, the boundaries between adipose tissue and the atrial wall remain indistinct. The best imaging modality to illustrate this architecture is cardiac magnetic resonance imaging (CMR), showing the septum in the same 2D format as 2D-TEE. CMR imaging clearly distinguishes muscle and adipose tissue (Figure 3B).

The FO can have different sizes and locations, depending on the variable extent of both the lamina tissue and the interatrial groove, which is not uniform throughout the FO (Figure 4). Defining the size of the FO is important because the safest place for a transseptal puncture (TSP) is through the FO. Given the wide variety of procedures in structural heart disease treated percutaneously, the site of the TSP (always within the FO) varies according to the procedure performed (site-specific TSP). For example, to implant a left atrial appendage occluder device in the correct position, the surgeon will puncture the septum posteriorly and inferiorly. In contrast, the position is higher for a mitral valve procedure (Figure 5A, 5B).

Generally, a larger FO is found in large atria, and a smaller FO has been associated with adipose tissue accumulation in the infolding, the so-called lipomatous hypertrophy (Figure 4C).

Another area of interest is the anterosuperior region, which abuts the transverse pericardial sinus and, through it, the aortic root, the most important structure close to the FO. A few millimeters separate the anterosuperior margin of the FO from the right atrial wall, which covers the non-coronary aortic sinus. In fact, the most feared complication of TSP is aortic root puncture (Figure 5C).

## Lipomatous hypertrophy

The term lipomatous hypertrophy of the IAS, coined by Prior in 1964, is questionable because, unlike lipoma, the lipomatous hypertrophy tissue is not encapsulated, and the accumulation of fat is histologically characterized by adipocyte hyperplasia (increased number), not hypertrophy (increased size). This accumulation of adipocytes is external to the heart, filling the interatrial groove. CMR images can define the external nature of this adipose tissue (Figure 3B). Although the accumulation of adipose tissue within the interatrial groove (SS) has a characteristic hourglass shape on echocardiography, both 2D and 3D-TEE (Figure 4) generally cannot clearly distinguish adipose tissue from the atrial walls. Hypertrophic edges were defined with an SS thickness of 8 mm, while lipomatous edges were defined with a thickness of 15 mm.

Lipomatous hypertrophy of the IAS is benign, although a large amount of adipose tissue may sporadically obstruct the entrance to the SVC or create distortion of the atrial walls with consequent arrhythmias.

## Septal Pouch and Ridge Septal

Anomalies in the IAS fusion can result in anatomical variants, including atrial septal pouch, left atrial septal ridge, and mixed anatomy, including pouch and ridge.^6^

Septal pouch, a kangaroo pouch-like structure without an interatrial shunt, opens more frequently in the LA or, less commonly, in the RA, probably occurring because of an incomplete fusion between the SP and SS (Figure 6A-6F).

**Figure 6:** – Transesophageal echocardiographic images showing a pouch-like structure in the IAS in two-dimensional (A) and three-dimensional (B) bicaval views with the orifice of the left septal pouch (curved arrows). (C and D) Color Doppler (C) and use of agitated saline microbubbles (D) showing the absence of septal shunt. Echocardiographic images t in (E and F) En face view of the IAS, from a left (E) and right (F) atrial perspective. Transesophageal echocardiographic images of the IAS with the presence of septal ridge. (G) Two-dimensional bicaval view showing septal ridge image (arrow). (H) Two-dimensional bicaval view showing IAS integrity during agitated saline contrast injection. (I) En face view (3D Zoom) of the IAS from a left atrial perspective showing three-dimensional echocardiographic morphology of the septal ridge (arrows). RA: right atrium; LA: left atrium; Asc Ao: ascending aorta, CS: coronary sinus, IAS: interatrial septum, IVC: inferior vena cava, SVC: superior vena cava, RUPV: right upper pulmonary vein.

In 2006, Roberson et al. described a malformation of the IAS consisting of a double IAS with a midline chamber.^7^ This description is believed to be consistent with a large septal pouch. It is still unclear, but it has been suggested that stagnant blood in this pouch may be a possible niche for thrombus formation and embolism.^8,9^ The 2D and 3D-TEEs are the best techniques to obtain images of the septal pouch.

The FO and the interatrial groove constitute the extensive area that covers the medial wall of the LA. Except for the septal pouch, this area is almost devoid of other structures. However, although rarely, a prominent membrane-like crest formation along the FO and extending to the atrial free wall has been observed.^10^ The atrial septal ridge, characterized by a tubular structure on the left atrial side of the IAS running along the FO, is a localized area of thickened tissue and is thought to be due to irregular fusion of the septa (Figure 6G-6I). A left atrial ridge may interfere with TSP in patients undergoing transcatheter procedures.

## Patent Foramen Ovale

PFO is not a true IAS defect, as there is no structural deficiency of the tissue, but a potential separation between the SP and SS, located in the anterosuperior portion of the IAS.^3^ The foramen remains functionally closed as long as the LA pressure is greater than the RA pressure and may only be functionally patent and have the appearance of a tunnel, with the SP forming an oscillating valve (Central Illustration, Figure 2). Relative differences in atrial pressures can result in intermittent shunting of blood. However, PFO can also be a true elliptical opening between the atria (Figure 7A-7C).

**Figure 7:** – Three-dimensional transesophageal echocardiographic en face view (3D Zoom mode) of the IAS, showing the FO lamina aneurysm associated with elliptical discontinuity (arrows) between the atria from left (A) and right (B) atrial perspective and confirmed with color Doppler (C). (D) Two-dimensional transesophageal image of stretched PFO (curved arrow) in bicaval view with color Doppler flow from left to right. (E, F) Two-dimensional transesophageal image of the IAS with excessive mobility of the FO lamina and (G) associated PFO (red arrow) demonstrated on color Doppler with flow from left to right and intermittent passage of agitated saline microbubbles from right to left (H). RA: right atrium; LA: left atrium; Ao: aorta; Asc Ao: ascending aorta; FO: fossa ovalis; CS: coronary sinus; IAS: interatrial septum; SVC: superior vena cava; IVC: inferior vena cava; MV: valve mitral; RUPV: right upper pulmonary vein.

Some PFO cases result from stretching the superior limbic band of the SS by atrial dilation and remodeling (Figure 7D). In other cases, the SP is truly aneurysmal and fails to completely close the ASD (Figure 7E-7H). The PFO size in autopsy studies ranges from 1 to 19 mm (mean 4.9 mm).^11^

For purposes of nomenclature, “PFO” is referred to as right-to-left blood flow demonstrated by color Doppler or saline contrast injection without a true IAS defect. “Left-to-right flow PFO” occurs when the atrial hemodynamics results from the potential opening of the foramen communication, resulting in a blood flow from left to right, as demonstrated by the Doppler image (Figure 7). When the PFO is stretched by atrial hemodynamics, it creates a septal defect, which is called a “stretched” PFO, and may result in left-to-right and/or right-to-left blood flow, depending on differences in the atrial pressures.

## Atrial Septal Aneurysm

Atrial septal aneurysm (ASA) is a saccular redundancy or deformity of the IAS, always limited to the FO lamina, and associated with increased mobility of the septal tissue. The best imaging modality to visualize ASA is 3D-TEE (Figure 8).

**Figure 8:** – Transesophageal echocardiographic images of the IAS. (A) Two-dimensional bicaval view showing a marked displacement of the FO lamina towards the RA, compatible with an aneurysm. (B, C) Three-dimensional en face view (3D Zoom) of the aneurysmal septum from the left and right atrial perspective, respectively. Two-dimensional transesophageal echocardiographic images of the interatrial septum. (D) Bicaval view showing the displacement of the FO lamina toward the LA, compatible with ASA. (E) M-mode of ASA demonstrating greater than 15 mm mobility of the FO lamina. RA: right atrium; LA: left atrium; ASA: atrial septal aneurysm; D: distance; FO: fossa ovalis; IVC: inferior vena cava; SVC: superior vena cava.

ASA is defined as a septal tissue (FO lamina) excursion ≥ 10 mm from the septal plane to the RA or the LA or a total combined left-right excursion of 15 mm. IAS excursion can be documented using 2D imaging as well as M-mode evaluation when the M-mode cursor is aligned perpendicular to the septal plane. This can be done in the bicaval view on TEE (Figure 8D, 8E).

The prevalence of ASA is 2% to 3%^12^ and has been associated with the presence and larger sizes of PFO and increased prevalence of cryptogenic cerebrovascular accident (CVA) and other embolic events and multiple septal fenestrations, which must be carefully evaluated using color Doppler imaging. The presence and extent of the ASA is a factor in selecting the PFO occluder device. A large device can be chosen to span and stabilize the IAS or a smaller one to better conform to the ASA.

## Eustachian Valve and Chiari Network

The Eustachian valve is the embryological remnant of the IVC valve that, in fetal life, directs the flow of the IVC through the FO, and extends from the junction between the IVC and the RA. It is considered prominent when its protrusion is ≥ 10 mm inside the RA. A large or prominent Eustachian valve in the context of PFO may indirectly contribute to paradoxical embolism, preventing the spontaneous closure of the foramen.^13^ When close to the IAS, it may interfere with the deployment of the RA side of the septal occluder device.

The Chiari network is a remnant of the right valve of the sinus venosus and appears as a mesh of filamentous structures in several places in the RA, including close to the mouth of the IVC and the coronary sinus. It is present in 2%-3% of the general population and is associated with the presence of PFO and ASA.^14^ The Chiari network can interfere with the passage of catheters and the device through the RA. Therefore, identifying the presence of the Chiari network should be part of the echocardiographic evaluation.

## Echocardiographic assessment of the interatrial septum

The 2D-TEE provides incremental anatomical information compared to transthoracic echocardiography (TTE) for IAS assessment; it also improves visualization of the IAS and surrounding structures. The IAS has a three-dimensional and dynamic anatomical structure, not being truly flat, with limitations in its evaluation using any form of 2D echocardiography. In addition, the 3D image offers unique views of the IAS and, in particular, allows for an “*en face*” or frontal view of the FO and its surroundings (Figure 2). Two-dimensional biplane (or triplane) imaging is a modality resulting from 3D technology, with the advantage of displaying and visualizing simultaneous echocardiographic images with a high frame rate and excellent temporal resolution (Figure 5A, 5B). A simultaneously displayed orthogonal image plane provides incremental information compared to a single plane imaging and is suitable for guiding transcatheter procedures.^15^

2D-TEE x IAS: Multiple, sequential views should be used for a complete and systematic assessment of the IAS, determining the size, shape, and location of any ASD and its relationship with surrounding structures. A sequential assessment from standard views with a gradual increase in transducer angle in a series of 15° increments is recommended to scan the ultrasound beam across areas of interest. Color Doppler is subsequently applied at a reduced scale at approximately 35-40 cm/s to identify low-velocity flows through a small fenestration, the PFO, or minor ASD. In TEE, five baseline views are used to assess the IAS: Short-axis upper esophagus, short-axis aortic valve mid-esophagus, four-chamber mid-esophagus, bicaval mid-esophagus, and long-axis mid-esophagus.

3D-TEE x IAS: 3D volumetric data are acquired in its limited sector presentations (live 3D), zoom, and full-volume from main views: short-axis mid-esophagus, bicaval, sagittal bicaval, and four chambers. When the IAS is viewed from the LA, it should be oriented with the right superior pulmonary vein at the 1 o’clock position. When viewed from the RA, the SVC should be located at the 11 o’clock position (Figure 2D, 2E).

## Echocardiographic Assessment of the Patent Foramen Ovale

TTE is used for the initial assessment of PFO. However, TEE is necessary for a more comprehensive evaluation of septal abnormalities due to better image quality. TEE is not invariably required to assess PFO if closure is not considered, but it should be performed in all patients assessed for percutaneous or surgical procedures.

TEE views used for PFO assessment begin in the mid-esophageal plane with settings optimized to view the IAS, and the echocardiographic image plane must be rotated, starting at 0° multiplane angles with 15° increments for complete septal evaluation. Low-scale color Doppler side-by-side imaging (35-40 cm/s) is useful to identify flow through the PFO and possible additional septal defects. The probe can be slightly withdrawn for a better assessment of the IAS close to the SVC and deepened for a better assessment of the IAS close to the IVC. From 30º-50º, with the AoV in cross-section, the PFO must be visualized adjacent to the aorta (Figure 7D, 7G, 7H). Rotating the imaging plane should align the PFO tunnel on the image. From this angle, the length of the tunnel can be assessed, and the SS^15^ thickness measured (Figure 9A-9F).

**Figure 9:** – (A) En face 3D-TEE view (TrueVue) of the elliptical PFO orifice. (B, C) 2D-TEE view showing PFO tunnel height increase after the Valsalva maneuver. (D, E) 2D and 3D-TEE view, respectively, with image plane adjustments for PFO tunnel measurement. (F) 2D-TEE view, adjusted to measure the IVC-PFO angle and measure the thickness and length of the SS. Transesophageal echocardiographic images during a percutaneous PFO occlusion procedure. (G) Bicaval view showing occluder device (*) well positioned, with flat disks and septal tissue between the disks. (H) 3D bicaval image confirming proper device positioning. (I) En face view from right atrial perspective showing the occluder device and adjacent structures. RA: right atrium; LA: left atrium; Asc Ao: ascending aorta; PFO: patent foramen ovale; SS: septum secundum; AoV: aortic valve; IVC: inferior vena cava; SVC: superior vena cava.

With the PFO visualized, saline contrast is injected to assess the right-to-left shunt. Important anatomical details of the IAS include the location of the PFO (anterior or superior region of the FO), the total length of the IAS, thickness and extent of the SS, length of the PFO tunnel, PFO size, the angle between the IVC and the PFO, the presence of ASA, and IAS fenestrations or defects (Figures 9A, 9B, 9C).

The 3D-TEE is used to define the PFO variations better and show its elliptical shape (Figure 9A). The PFO area changes during the cardiac cycle and is larger during ventricular systole. The 3D-TEE is also used to guide the closure procedure with “*en face*” views of the IAS, showing the PFO’s and device’s relationship with adjacent structures (Figure 9G-9I).

Specific anatomic features of the PFO must be evaluated when deciding on the occluder device. Specifically, PFO tunnel length, ASA presence and size, SS thickness, and maximum PFO size during the cardiac cycle are important aspects to be considered when selecting the correct device and enable a lower incidence of complications compared to the single-device-to-all-PFO strategy.^16^

## Shunting Characteristics

Color Doppler can detect a shunt through the PFO. However, the shunt is often intermittent and may not be easily detectable. When the PFO is stretched by pressure differences in the LA and AD, the left-to-right shunt can be seen on color Doppler (Figure 7). Contrast echocardiography using agitated saline macrobubbles (MB) combined with physiological maneuvers increases the sensitivity of PFO detection.^17,18^ Agitated macrobubbles are too large to pass normal pulmonary vasculature and are easily detected by echocardiographic imaging because of their increased echogenicity (Figure 7H). Contrast-enhanced TTE can be used to detect PFO with reasonable sensitivity and specificity. Nevertheless, TEE is considered the gold standard. Whether using TTE or TEE, a standardized protocol improves test accuracy, as shown below. The protocol includes multiple saline injections and provocative maneuvers to increase RA pressure, such as the Valsalva maneuver (VM), coughing, or abdominal compression.^19,20^

Protocol (Supplementary Figure 1):

- Insert an intravenous catheter into the antecubital vein, connected to a three-way stopcock.
- In a 10 mL syringe connected to the stopcock, mix 8 mL of saline solution with 1 mL of the patient’s blood and 1 mL of air. Adding blood to the contrast solution results in increased intensity of the macrobubbles detected by echocardiography.^20^
- Rapidly agitate the solution back and forth between two 10 mL syringes attached to the three-way stopcock to produce bubbles.
- Inject the rapidly agitated solution into the antecubital vein along with the echocardiographic acquisition of a long image clip (about 10 beats), recorded from the four-chamber view on TTE, and the best view of the IAS on TEE is usually between 30º-100º.
- Biplane imaging can improve the detection of small right-to-left blood flows.

The appearance of macrobubbles in the LA within 3-6 heartbeats after RA opacification is considered positive for the presence of an intracardiac shunt. Ideally, the bubbles will be visualized crossing the IAS through the PFO (Figure 10E, 10G).

**Figure 10:** – Transesophageal echocardiographic images (A, B, C, D) show two-dimensional views of saline microbubbles entering the LA from the right pulmonary veins in a patient with pulmonary arteriovenous fistula. (E) Bicaval two-dimensional view showing microbubbles crossing the atrial septum through the FO, confirming the presence of PFO. (F, G, H) Three-dimensional en face views of left atrial perspective, confirming the arrival of bubbles in the LA (arrow) through the PFO. RA: right atrium; LA: left atrium; FO: fossa ovalis; PFO: patent foramen ovale; RLPV: right lower pulmonary vein; RUPV: right upper pulmonary vein.

Physiological maneuvers that transiently increase RA pressure are required for the right-to-left passage of macrobubbles to identify PFO when no shunt is present. The VM is commonly performed and must be maintained for enough time for the bubbles to fill the RA; its effectiveness can be assessed via echocardiography by the presence of IAS bulging to the left, indicating higher pressure in the RA compared to the LA. The appearance of macrobubbles in the LA after 3-6 heartbeats indicates an intrapulmonary shunt, such as an arteriovenous malformation, which is confirmed when bubbles are visualized entering the LA from the pulmonary veins and not visualized crossing the IAS (Figure 11A-11D). Other reasons for false-positive bubble studies for PFO are sinus venosus septal defect or other unidentified ASD.

Bubble studies may result in false-negative findings due to inadequate RA opacification, inadequate VM, presence of Eustachian valve directing venous return from the IVC to the IAS (preventing MB from the SVC from crossing the septum), inability to increase RA pressure above LA pressure, as in left ventricular diastolic dysfunction, and poor image quality. With this quality, second harmonic imaging can improve the identification and detection of macrobubbles. Rarely, an injection into the leg vein can also be used to bypass the very large Chiari network or Eustachian valve that ends up preventing the bubbles from filling the RA from the SVC. The right-to-left shunt can be quantified based on the number of bubbles that appear in the left heart in a static echocardiographic image. However, this number depends on the amount of injected macrobubbles and an adequate VM.

## Echocardiography in transcatheter closure

Echocardiography is used to aid percutaneous PFO closure, providing significant information for patient and device selection, guiding the procedure, monitoring complications, and evaluating results. The choice of the occluder device should consider the anatomy of the IAS and PFO, device availability, and staff experience. The choice usually favors devices that are specifically recommended for closing this structure. However, in specific cases, due to the analysis of the anatomy, a device designed for ASD occlusion can be chosen.^21^

In October 2016, the US Food and Drug Administration (FDA) approved the use of the Amplatzer PFO occluder (St. Jude Medical, Inc.) for percutaneous closure of PFO and became the first commercially available device in the United States for use in patients with presumably PFO-mediated cryptogenic stroke. The three devices studied in the United States in large randomized controlled trials are Amplatzer PFO occluder, StarFlex septal occluder (NMT Medical, Inc.), and Helex/Cardioform septal occluder (Gore & Associates).

## Device embolization and erosion

Complications of PFO occluder devices are rare and include device embolization, cardiac perforation, tamponade, and erosion.^22^

Device embolization occurs in approximately 0.1%–0.4% of cases and is more common with ASD closure devices.^22^ It is potentially fatal and requires immediate removal by percutaneous or surgical intervention and is diagnosed by routine TTE, which assesses the location of the displaced device and physiological sequelae (inflow/outflow obstruction, valve rupture) resulting from embolization.

Device erosion is rare but potentially fatal, can occur with multiple devices, reaches the roof of the RA or LA or aortic junction, and can result in hemopericardium, tamponade, aortic fistula, and/or death.^23^ Erosion can begin as a subclinical event, with the device colliding with surrounding structures, straining the atrial or aortic tissue, or resulting in subclinical pericardial effusion. Most erosions occur in the first week after implantation.^24^ The most significant factors for this complication are device oversizing (40% of the cases), complete absence of aortic rim, high/upper septal location of the defect, and associated insufficiency of the posterior rim. Important risk factors for erosion after device placement include deformation of the closure device at the aortic root and pericardial effusion observed within 24 hours of implantation.

IAS imaging immediately after the procedure

An echocardiographic evaluation is performed to confirm that the right and left disks of the occluder device are flat and apposed to the septum with septal tissue between the disks. The device, IAS, and surrounding structures are assessed before release (Figure 9G-9I). A color Doppler evaluation is performed to exclude residual flow at the device’s margins, which suggests improper device size or position. Interference with pulmonary veins, coronary sinus, atrioventricular valve function, and aortic root deformation are carefully evaluated and excluded before release.

## Patent Foramen Ovale and cryptogenic stroke

Cryptogenic stroke is defined as cerebral infarction not attributable to a defined source of cardioembolism, large artery atherosclerosis, small artery disease, or other determined etiologies such as non-atherosclerotic vasculopathies, hypercoagulable states or hematological disorders despite extensive vascular, cardiac, and serologic evaluation – TOAST criteria (Trial of Org 10172 in Acute Stroke Treatment).^25^ It affects about 25% to 40% of patients with cerebrovascular ischemia.^26^

Embolic stroke of unknown origin is a subcategory of cryptogenic ischemic stroke that specifically denotes non-lacunar stroke with no immediately identifiable etiology.^27^

PFO has been associated with multiple pathologies: migraine, platypnea-orthodeoxia syndrome, high altitude decompression sickness and, most commonly, cryptogenic stroke.^28-30^

The association between cryptogenic stroke and PFO was first credited to pathologist Julius Cohnheim in 1877. However, modern interest began after Lechat et al. observed in 1988 that PFO was present in 40% of individuals with cryptogenic stroke compared to only 10% in controls.^31^ Since then, other studies have documented the association of PFO with patients who suffer from stroke, particularly aged < 55.^32^

POF has been associated with cryptogenic stroke due to paradoxical embolism, in which a clot or embolic particle from the peripheral venous circulation passes into the arterial circulation through a right-to-left shunt, bypassing the natural filtration system in the pulmonary vasculature or even thrombosis *in situ* in the PFO tunnel, local blood stasis on the left side of the IAS, or thrombus formation within the ASA concomitant with the PFO, and indirectly through increased arrhythmia susceptibility. There are indications that the electrical activity of the LA can be altered in the presence of PFO, which further increases the risk of stroke. IAS stretching induced by septal aberrations (large PFO tunnel, ASA) can modify atrial depolarization, activating arrhythmogenic substrate.^33^ Thrombi crossing the PFO can occasionally be observed both in autopsies and in a few reports of echocardiographic examinations (Supplementary Figure 2), suggesting this mechanism is a possible cause of paradoxical embolism (Central Illustration).

Despite the infrequent visualization of thrombi in PFO, epidemiological observation indicates that it is responsible for a considerable number of strokes. History of deep vein thrombosis, pulmonary embolism, pulmonary arterial hypertension, prolonged travel, VM preceding the onset of stroke symptoms, migraine, and sleep apnea have been described as independent risk factors for the association between PFO and stroke.^34^ Other mechanisms, such as undetected or occult atrial fibrillation (AF), may be responsible for cryptogenic stroke. Occult AF has been associated with a 2.5-fold increase in the risk of stroke, although with a low temporal relationship.^35^ Likewise, mitral valve disease, spontaneous contrast in the LA, calcification of the mitral annulus, cardiac prostheses, cardiac tumors, aortic atheroma, and blood disorders with susceptibility to thrombus formation.^36^ A minimal work-up is proposed for stroke of undetermined origin with tomography/magnetic resonance of the skull, echocardiogram, extra/intravascular imaging, research for hypercoagulable disorders, and cardiac monitoring for ≥ 24 hours.^37,38^ Cardiac monitoring is important to unmask occult arrhythmias, specifically AF, which is still the most common cause of cryptogenic stroke.

TTE and, especially, TEE using saline bubbles are part of the PFO’s assessment, including shunt detection, anatomical characteristics of the PFO, differential diagnosis of other atrial septal defects, or pulmonary shunt.^39^

Transcranial Doppler is more sensitive but less specific than TEE in diagnosing PFO. Its insensitivity to making a differential diagnosis between cardiac and pulmonary shunt and the impossibility of diagnosing PFO anatomical changes justify its lower specificity.^40^ However, there is controversy about the ideal management of PFO in cryptogenic stroke. The main treatment was antithrombotic therapy, derived from the FRENCH PFO-ASA^41^ and PICSS^42^ studies. These did not show an increased risk of stroke recurrence, regardless of the presence of PFO. Despite the embolic hypothesis of cryptogenic stroke in PFO, anticoagulation was not superior to aspirin in preventing recurrent strokes. These findings were also replicated in the NAVIGATE ESUS^43^ study, which found a non-statistically significant reduction in ischemic stroke with anticoagulation compared to aspirin. However, the reported recurrence rate in cryptogenic stroke is substantial and can range from 3% to 6%.^37^ Therefore, mechanical treatment options for PFO should be considered. Surgical closure of the defect resulted in significant morbidity and no evidence of benefit in preventing recurrences.^44^

## Evidence for percutaneous PFO closure

Percutaneous PFO closure is associated with significantly less morbidity than surgery and is an option in paradoxical embolism. Over the last decade, the challenge has been to demonstrate the benefit in populations with a low incidence of stroke. Two main trials, CLOSURE I^45^ and PC trial,^46^ failed to demonstrate a clear superiority of percutaneous closure over drug therapy. There was, however, an increased incidence of atrial arrhythmias in the PFO device closure group. Next, the RESPECT study^47^ showed that PFO closure with the Amplatzer PFO Occluder was superior to drug management in reducing long-term recurrent ischemic stroke in patients with cryptogenic stroke and evidence of PFO. Based on cumulative trial data, the 2014 AHA/ASA guidelines provided a Class III recommendation for PFO closure in patients with cryptogenic stroke without evidence of deep vein thrombosis.^48^

However, early trials had significant limitations. They used older devices associated with higher complication rates and follow-up time averaging only 2-4 years. Observational trials have reported favorable long-term outcomes.^49,50^ Additionally, patient meta-analyses have demonstrated the superiority of using the Amplatzer PFO Occluder Device.^51,52^

Subsequently, three additional randomized clinical trials, CLOSE,^53^ DEFENSE-PFO,^54^ and REDUCE,^55^ showed superiority of percutaneous PFO closure compared to drug therapy alone.

Multiple meta-analyses have assessed evidence of PFO closure. Nasir et al. investigated the six main trials evaluating PFO closure: CLOSURE I, PC trial, RESPECT, CLOSE, REDUCE, and DEFENSE-PFO, with a total of 3,510 patients. This study showed the persistent benefit of PFO closure versus antiplatelet or anticoagulation alone, with the number needed to treat being 41 (OR 0.34, 95% CI 0.15–0.79, p = 0.012). Major side effects were comparable, with no difference in mortality or major bleeding events.^56^ Wintzer-Wehekind et al. demonstrated that, with percutaneous PFO closure and after discontinuation of antiplatelet therapy, there was no increase in events in long-term follow-up.^57^ (Supplementary Table 1) shows the main randomized studies in this setting.

Several studies have also sought to determine, with greater precision, which patients would most benefit from interventional treatment. Data from recent studies suggest that, while associated with the ASA, larger PFO and hypermobile septum could obtain greater benefits from closure. TEE was used as an imaging modality to assess PFO in all the trials discussed. While there were no recommended cut-off values for use in these trials, CLOSE only included PFO with > 30 bubbles (MB) or associated ASA > 10 mm, and REDUCE stratified shunt size into four groups (0 MB without shunt; 1–5 MB Small; 6–25 MB Moderate; > 25 MB Large), 81% with moderate or large shunts.

Despite the positive results of PFO closure data, there are limitations. In five of the six studies, the age limit was set at 60, with the remainder being insufficient for those over 60. Therefore, the results cannot be generalized to the entire population.^56^ There was a 4.7% increase in the risk of new AF in the closure group compared to the drug treatment group.^58^ This can be attributed to atrial irritation during the procedure, as most events were periprocedural and transient. Additionally, the procedure has rare complications, including atrial perforation with tamponade requiring surgical removal of the device. Finally, there is the potential long-term risk of root dilation and subsequent erosions caused by the device,^59^ as well as potential thrombus formation in the device.^60^

## Candidates for PFO Closure

Despite evidence linking PFO and cryptogenic stroke, not all PFO needs to be closed. About one-third of PFOs are found incidentally, with other more plausible stroke etiologies.^50^ If conventional investigation does not reveal the cause of the stroke, screening the patient for percutaneous PFO closure, according to patient-specific risk factors, is justified.

The Risk of Paradoxical Embolism (RoPE) Score was created to help identify stroke patients with PFO in whom PFO is the likely cause. (According to Supplementary Table 2).

This index was created using regression data from 12 databases involving patients with cryptogenic stroke^61,62^ and considers clinical data such as history of hypertension, diabetes, stroke or transient ischemic attack (TIA), smoking, cortical infarction evidenced in imaging exams, and patient’s age. The higher the score, the more likely it is that PFO is the cause of the cryptogenic stroke (Supplementary Table 3).

Scores of 7, 8, and 9-10 correspond, respectively, to a 72%, 84%, and 88% chance that PFO is the cause of cryptogenic stroke and define a subset of patients who may benefit from closure. In addition, a low RoPE score ≤ 6 was identified as an independent risk factor for mortality and recurrent ischemic stroke after PFO closure, along with other risk factors such as larger left atrial dimensions.^63^ Young patients with superficial stroke and without vascular risk factors have a high score. In low-score patients, elderly patients with vascular risk factors, the presence of PFO suggests that it is accidental. The risk of stroke or TIA is calculated at two years for each group.

In a recent observational study, 107 patients with and without cryptogenic stroke who were scheduled for percutaneous PFO closure had their anatomical features evaluated by TEE. The findings demonstrated that some PFO anatomical features predispose to the formation and passage of thrombi from the RA to the LA, resulting in systemic embolisms.^64^

The evaluated echocardiographic characteristics of PFO were:

- **PFO height:** maximum separation between the SP and SS in the end-systolic frame. Considered large when ≥ 2.0 mm.
- **Tunnel Length:** Maximum overlap between the SP and SS. Length ≥ 10 mm was defined as a long tunnel PFO, which can be a space for thrombus formation.
- **Degree of Right-to-Left Shunt:** assessed at rest and after VM using saline contrast. The number of MB is counted in a single frame, and > 20 MB is considered a large shunt with a higher risk of stroke.
- **Angle between the IVC and PFO Flap:** measured in the image plane that displays the IVC and the IAS. A low PFO angle is defined as ≤ 10º, which can preferentially direct the blood flow from the IVC to the IAS and the PFO orifice, posing an increased risk for stroke.
- **Interatrial Septal Aneurysm:** redundant, mobile tissue in the FO region, with midline phasic septal excursion to the RA or LA ≥ 10 mm or total excursion ≥ 15 mm between the RA and the LA.
- **Hypermobility of the Interatrial Septum:** mobile septum with excursion ≥ 5 mm in each heartbeat. The mechanisms that have been proposed as responsible for paradoxical embolism caused by aneurysm or IAS hypermobility are:

1. Paradoxical embolism due to the passage of a thrombus from the RA to the LA through the PFO.
2. ASA without an intracardiac shunt, small thrombi of fibrin and platelets may form on the left side of the septum, detaching due to oscillation of the aneurysm.

- **Eustachian Valve and Chiari Network:** they can direct the flow, which comes through the IVC directly to the IAS, favoring PFO and ASA and, indirectly, facilitating paradoxical embolism. PFO with a large right-to-left shunt was seen more frequently in patients with a Chiari network.^65^

Echocardiographic features that increase the risk of stroke include large PFO size, large right-to-left shunt, spontaneous right-to-left shunt, greater mobility of the PFO lamina, prominent Eustachian valve, Chiari network, and ASA. The multivariate analysis of these observations showed echocardiographic factors that were independent predictors of ischemic cerebral events shown in Supplementary Table 4.

Based on the anatomical data analyzed by echocardiography, a risk score was created for POF, which is responsible for paradoxical embolism in stroke patients (Supplementary Table 5).

Elevated scores are observed in young patients with superficial stroke without or with few traditional risk factors. Patients with low scores, those with vascular risk factors and the elderly, suggest that PFO is accidental and not directly responsible for the ischemic event. The risk of stroke/TIA is calculated for two years.

Findings from the CLOSE and DEFENSE-PFO studies support these observations. Studies that included only high-risk PFO demonstrated no recurrent stroke in the closure arm at five and two years, respectively. In contrast, short tunnel PFO has also been reported in patients with ischemic stroke.^66^ Thus, further studies are needed to elucidate the relationship between PFO tunnel length and stroke, identifying the mechanisms.

Determining shunt severity by the number of bubbles crossing the IAS through the PFO may not be completely accurate, as some bubbles may not be in the imaging plane. There is likely to be little difference between the shunt with 20 and 21 bubbles, but in this analysis, one is classified as small and the other as large. Furthermore, dichotomous determination of shunt severity (small vs. large) may be prone to significant bias. The VM substantially depends on the patient’s effort, and the lack of consistency can affect the morphometric characteristics of the PFO.

## Conclusions

In recent years, the influx of evidence showing the benefit of percutaneous PFO closure in cryptogenic stroke compared to medical therapy alone and the evolution of percutaneous procedures involving structural heart disease and electrophysiological procedures have brought additional interest in the anatomy of the IAS. Based on current data, a thorough patient assessment and a detailed and standardized anatomical characterization of the IAS are recommended.

**Figure 5:** – Biplane 3D-TEE images (X-plane) guiding the site-specific atrial transseptal puncture for percutaneous occlusion of the left atrial appendage in a more posterior and inferior position of the septum. The arrow indicates a well-placed puncture catheter. (C) En face 3D-TEE image (right atrial perspective) of the IAS showing the proximity (double arrow) of the non-coronary aortic sinus to the FO region. Aortic sinus and proximal portion of the ascending aorta are outlined. RA: right atrium; LA: left atrium; Asc Ao: ascending aorta; FO: fossa ovalis; NC: non-coronary aortic sinus; AoV: aortic valve; IVC: inferior vena cava; SVC: superior vena cava.
